# The granulosa cell response to luteinizing hormone is partly mediated by YAP1-dependent induction of amphiregulin

**DOI:** 10.1186/s12964-022-00843-1

**Published:** 2022-05-26

**Authors:** Philippe Godin, Mayra F. Tsoi, Martin Morin, Nicolas Gévry, Derek Boerboom

**Affiliations:** 1 Centre de Recherche en Reproduction et Fertilité (CRRF), Université de Montréal, Saint-Hyacinthe, QC Canada; 2 Department of Biology, Université de Sherbrooke, Sherbrooke, QC Canada

**Keywords:** Yap1, Hippo pathway, Amphiregulin, Luteinizing hormone, Granulosa cells, Ovulation

## Abstract

**Background:**

The LH surge is a pivotal event that triggers multiple key ovarian processes including oocyte maturation, cumulus expansion, follicular wall rupture and luteinization of mural granulosa and theca cells. Recently, LH-dependent activation of the Hippo signaling pathway has been shown to be required for the differentiation of granulosa cells into luteal cells. Still, the precise interactions between Hippo and LH signaling in murine granulosa cells remain to be elucidated.

**Methods:**

To detect the expression of effectors of the Hippo pathway, western blot, immunohistochemical and RT-qPCR analyses were performed on granulosa cells treated with LH in vitro or isolated from immature mice treated with eCG and hCG. Cultured granulosa cells were pretreated with pharmacologic inhibitors to identify the signaling pathways involved in Hippo regulation by LH. To study the roles of *Yap1* and *Taz* in the regulation of the LH signaling cascade, RT-qPCR and microarray analyses were done on granulosa cells from *Yap1*^f/f^;*Taz*^f/f^ mice treated with an adenovirus to drive cre expression. RT-qPCR was performed to evaluate YAP1 binding to the *Areg* promoter following chromatin immunoprecipitation of granulosa cells collected from mice prior to or 60 min following hCG treatment.

**Results:**

Granulosa cells showed a transient increase in LATS1, YAP1 and TAZ phosphorylation levels in response to the ovulatory signal. This Hippo activation by LH was mediated by protein kinase A. Furthermore, Yap1 and Taz are required for the induction of several LH target genes such as *Areg*, *Pgr* and *Ptgs2*, and for the activation of the ERK1/2 pathway. Consistent with these results, there was a substantial overlap between genes that are upregulated by LH and those that are downregulated following loss of *Yap1*/*Taz,* highlighting a major role for Hippo in mediating LH actions in the ovulation process. Finally, we showed that there is a marked recruitment of YAP1 to the *Areg* promoter of granulosa cells in response to hCG stimulation.

**Conclusions:**

Overall, these results indicate that Hippo collaborates with the cAMP/PKA and ERK1/2 pathways to participate in the precise regulation of the LH cascade, and that *Areg*, as a direct transcriptional target of YAP1, is involved in mediating its actions in the ovary.

**Video Abstract**

**Supplementary Information:**

The online version contains supplementary material available at 10.1186/s12964-022-00843-1.

## Background

In the cycling mammalian female, folliculogenesis culminates with a luteinizing hormone (LH) surge that triggers ovulation. This singular event is a pivotal point for the fate of most ovarian cells, as it leads to resumption of oocyte meiosis, cumulus expansion, follicular wall rupture and luteinization of mural granulosa cells (GCs) and theca cells. Failure in any of these processes can lead to ovulation defects, which prevent fertilization and impair female reproductive success. Binding of LH to its G-protein-coupled receptor (LHCGR) results in an increase in intracellular levels of cAMP, leading to the activation of PKA and CREB. Although they respond to the LH surge, the oocyte and cumulus cells do not express *Lhcgr*. Instead, key components of the epidermal growth factor (EGF) family, notably amphiregulin (*Areg*), epiregulin (*Ereg*) and betacellulin (*Btc*), secreted by mural GCs following PKA-dependent CREB phosphorylation, mediate the paracrine transmission of the LH signal [1, 2]. Subsequent EGF receptor (EGFR) activation leads to sustained activation of the extracellular signal-regulated kinases 1 and 2 (ERK1/2, also known as mitogen-activated protein kinases 3 and 1 [MAPK3/1]) [3], which is essential for LH-induced oocyte maturation, cumulus expansion and luteinization [4–6]. Even though the involvement of the PKA/CREB, EGFR and ERK1/2 pathways in the LH cascade has long been recognized, the precise mechanisms of LH action in GCs remain incompletely understood.

The Hippo pathway is a master regulator of cell proliferation and differentiation in a wide variety of cell types. It is an evolutionarily conserved signaling pathway well-known for its central role in embryo development, organ size determination and carcinogenesis. Activation of the canonical Hippo pathway by various upstream signals ultimately results in the phosphorylation of yes-associated protein 1 (YAP1, at serine residues S127 and S397) and transcriptional coactivator with PDZ-binding motif (TAZ, at S89) by large tumor suppressor kinases 1 and -2 (LATS1/2) [7]. Phosphorylation at these serine residues promotes the cytoplasmic retention and/or ubiquitin-dependent degradation of YAP1/TAZ [8–11], which prevents their binding to transcription factors located in the cell nucleus (notably those of the TEAD and RUNX families [7]), and thereby prevents the transcription of Hippo target genes [12]. Notable YAP1/TAZ transcriptional target genes include members of the CCN family of matricellular proteins (*Ccn1, 2*, *3*, *5*, *6*), members of the baculoviral inhibitors of apoptosis repeat containing (*Birc5, 7)* family and EGF family member *Areg* [7].

Although early research on Hippo signaling was mainly focused on organ and tumor development, its roles in the physiology of normal adult tissues have been gaining increased attention. In the ovary, inactivation of the Hippo pathway has been shown to be required for GC proliferation. Ovarian fragmentation leads to a YAP1-dependent activation of follicular development [13]. In human GC tumor cells and in bovine GCs, loss of *Yap1* and/or *Taz* expression negatively affects proliferation and steroidogenesis [14, 15]. Likewise, conditional inactivation of *Yap1* in GCs at all stages of folliculogenesis in mice resulted in fewer corpora lutea and reduced fertility [16]. Recent reports have also suggested that Hippo is activated in GCs in response to the LH surge. Following the ovulatory signal, YAP1 is phosphorylated and exported from the nucleus of GCs [16–18]. In addition, YAP1 overexpression in GCs has been shown to prevent adequate induction of LH target genes [17] and progesterone production [16]. The Hippo pathway therefore appears to be a negative regulator of follicle growth, but its activation by LH may be required for LH to exert its effects during terminal follicle differentiation.

As *Areg* has been shown to be a direct transcriptional target of Hippo in human epithelial breast [19] and keratinocyte [20] cell lines, in this study, we investigated the interactions between the Hippo pathway and the LH signalling cascade, with emphasis on the potential regulation of *Areg* by Hippo in GCs. Here we show that Hippo activation following the ovulatory signal is a PKA-dependent process. Knockdown of *Yap1* and *Taz* expression in primary cultures of GCs demonstrates their requirement for the induction of a number of genes involved in biological processes related to ovulation. Finally, we report for the first time the direct transcriptional regulation of *Areg* by the Hippo pathway in murine GCs.

## Methods

### Animal models, ovary collection and GC isolation

C57BL/6 J wild-type (WT) mice were purchased from The Jackson Laboratory. Mice bearing floxed alleles for *Yap1* and/or *Taz* (referred to as *Yap1*^f/f^, *Taz*^f/f^ and *Yap1*^f/f^;*Taz*^f/f^) were graciously provided by Eric Olson (University of Texas Southwestern Medical Center). Genotyping analyses were performed on DNA extracted from tail biopsies as previously described [21–23]. Immature (22- to 25-day old) female mice were treated with 5 IU of equine chorionic gonadotropin IP (eCG; Intervet Canada Corp.), followed or not 48 h later by an ovulatory dose of 5 IU of human chorionic gonadotropin IP (hCG; Intervet Canada Corp.). Whole ovaries obtained 48 h after eCG and 4 h after hCG were fixed in 10% formalin for immunohistochemical analyses. Ovaries collected 48 h after eCG and 1, 4, 8 or 12 h after hCG were placed in HBSS, and their follicles were punctured using 26-gauge needles to release GCs. Cells were then centrifuged (2000 g, 10 min) without any additional filtration step, HBSS was removed, and GCs were flash frozen for immunoblotting (n = 4 mice/time point) or real-time PCR (RT-qPCR) (n = 4 mice/time point, done three times), or cross-linked for chromatin immunoprecipitation (ChIP) analyses (n = 4 biological replicates/time point, one replicate represents the GCs of 4 mice), as described below.

### Cell culture

Isolated GCs from eCG-stimulated immature WT mouse ovaries were seeded onto 96-well plates (ThermoFisher Scientific) at a density of 0.5 ovaries per well in MEM 1X (ThermoFisher Scientific) supplemented with 0.25 mM sodium pyruvate (ThermoFisher Scientific), 3 mM L-glutamine (Wisent Inc.), penicillin–streptomycin (Wisent Inc.) and 1% fetal bovine serum (FBS; Wisent Inc.) for 3 h at 37 °C. Cells were serum starved for 2 h before treatment with 50 ng/ml human recombinant LH (National Hormone & Peptide Program) or PBS (control) for 5, 15, 30 or 60 min. Alternatively, cells were pretreated with inhibitors against PKA (H-89; 10 μM for 30 min; Tocris and PKA inhibitor 14–22 amide [PKI]; 50 μM for 30 min; MilliporeSigma), AKT1/2/3 (MK-2206; 10 μM for 60 min; Selleckchem) or MEK1/2 (U0126; 10 μM for 60 min; Selleckchem) prior to treatment with LH for 30 min. Isolated GCs from *Yap1*^f/f^, *Taz*^f/f^ and *Yap1*^f/f^;*Taz*^f/f^ eCG-primed immature mice were seeded onto 96-well plates (0.5 ovaries/well) as described above, but supplemented with 2% FBS for 4 h before infection with either Ad5-CMV-eGFP (Ad-eGFP; eGFP control adenovirus; Vector Biolabs) or Ad5-Cre-eGFP (Ad-Cre; Cre recombinase adenovirus; Vector Biolabs) for 18 or 24 h in 2% FBS using a multiplicity of infection of 50. After infection, cells were serum starved for 2 h prior to treatment with LH for 30 min, 1, 2 or 6 h or with 10 μM forskolin (FSK; Selleckchem) for 2 h. For all of the experiments described above, cells were harvested for subsequent immunoblotting, RT-qPCR or microarray analyses.

### Immunohistochemistry

Immunohistochemical (IHC) analyses were performed on formalin fixed, paraffin-embedded, 3 μm thick ovarian sections. Following deparaffinization, rehydration and sodium citrate heat-mediated antigen retrieval, sections were incubated with Phospho-LATS1(Thr1079) [24] or Phospho-YAP1(S127) [25] antibodies at a 1:500 dilution overnight at 4 °C. Detection was performed using the Vectastain Elite ABC HRP Kit followed by the DAB Peroxidase (HRP) Substrate Kit according to the manufacturer’s instructions (Vector Laboratories). Slides were counterstained with hematoxylin prior to mounting. Negative controls underwent the same steps but were incubated with the diluent alone without the primary antibody.

### Western blot analyses

Proteins were extracted from freshly isolated and cultured GCs by lysis in SDS loading buffer containing 5% β-Mercaptoethanol (BioShop). Samples were resolved on 10% SDS–polyacrylamide gels and transferred onto Immobilon-PSQ PVDF membrane (MilliporeSigma). Membranes were blocked with 5% non-fat dry milk in Tris-buffered saline supplemented with 0.1% Tween 20 (BioShop)(TBST) and sequentially probed with antibodies raised against Phospho-LATS1(Thr1079) [24], LATS1 [26], Phospho-YAP1(S127) [25], Phospho-YAP1(S397) [27], YAP1 [28], Phospho-TAZ(S89) [29], TAZ [30], Phospho-AKT(S473) [31], AKT [32], Phospho-p44/42 MAPK(Erk1/2)(Thr202/Tyr204) [33], p44/42 MAPK(Erk1/2) [34], Phospho-CREB(S133) [35] and/or CREB [36] at a 1:1000 dilution overnight at 4 °C in TBST supplemented with 5% BSA (Bioshop). Membranes were then probed with anti-rabbit IgG HRP Conjugate (Promega) [37] diluted in 5% non-fat powdered milk for 1 h at room temperature. When required, membranes were stripped and blocked with 5% non-fat dry milk in TBST before reprobing. Stripping involved two 5 min incubations of stripping buffer (0.2 M glycine [Bioshop]; 0.1% sodium dodecyl sulfate [SDS—MilliporeSigma]; 1% Tween 20; pH adjusted to 2.5), PBS and TBST. The antibody against β-actin (loading control) [38] was diluted 1:10 000 in 5% non-fat powdered milk and incubated for 30 min at room temperature. Immunosignal was detected with Immobilon Western Chemiluminescent HRP substrate (MilliporeSigma). The images were captured with ChemiDoc MP Imaging System (Bio-Rad) and analyzed with Image Lab Software v.5.0 (Bio-Rad).

### Microarray analyses and bioinformatics

Total RNA extracted from cultured *Yap1*^f/f^/*Taz*^f/f^ GCs infected either with Ad-Cre for 18 h (or Ad-eGFP as a control) or from WT GCs treated with LH for 3 h (or PBS as a control) was submitted for triplicate microarray analyses after quality control assessment. The Affymetrix Mouse Clariom S Assay (ThermoFisher) was used and all steps were performed by the McGill University and Génome Québec Innovation Centre. Data were pre-processed using the Affymetrix Gene Expression Console software (ThermoFisher), and differential analysis of gene expression was done by the R package limma followed by a Student’s t-test. Microarray data have been deposited in the GEO database under accession number GSE184396. Raw data from differentially expressed genes (DEGs) in *Erk1*/*2*-deficient GCs were graciously provided by Joanne S Richards and Heng-Yu Fan (Baylor College of Medicine) [4]. Cut-off thresholds of *P* ≤ 0.05 and |FC|≥ 1.5 were used to identify DEGs. Overlapping DEGs were identified using a Venn diagram online tool (http://bioinformatics.psb.ugent.be/webtools/Venn/). Oocyte-specific genes were removed from the overlapping DEG lists, as their presence was presumably caused by oocyte contamination of our primary GC cultures. Gene Set Enrichment Analysis of the overlapping DEGs was performed using either Panther [39] (www.pantherdb.org), Metascape [40] (www.metascape.org) or DAVID [41] (www.david.ncifcrf.gov) bioinformatics resources. DNA-motif enrichment in the *Areg* promoter was performed using the peak annotation function of the HOMER software for motif enrichment (v4.11) (www.homer.ucsd.edu/homer/).

### Chromatin immunoprecipitation (ChIP)

ChIP experiments were performed using isolated GCs from eCG-primed immature WT mice before or after 60 min of hCG stimulation as described above. 10% of each sample was kept in a separate tube for further assessment of sample quality. Remaining GCs were cross-linked with 1.2 ml of 1.1% formaldehyde in PBS at room temperature with agitation. Fifteen minutes later, the cross-linking reaction was stopped by adding 0.125 M glycine for 5 min. Cells were then centrifuged (1500 g, 5 min, 4 °C) and gently resuspended in cold PBS (4 °C) twice before being flash-frozen. Cross-linked GCs were first subjected to a nucleus extraction step based on the NEXSON method described in Arrigoni [42]. Briefly, each sample was resuspended in 500 μl of nucleus isolation solution (10 mM Tris–HCl pH 8; 10 mM NaCl; 0.2% Igepal CA-630 [MilliporeSigma]; protease inhibitors) and then sonicated for 45 s at low intensity using the BioRuptor 300 Sonicator (Diagenode). After centrifugation (1000 g, 5 min, 4 °C), pelleted nuclei were lysed in 100 μl of ChIP SDS lysis buffer for 60 min on ice. Lysed nuclei were sonicated for 9 min at high intensity and the resulting chromatin solution was diluted tenfold in ChIP dilution buffer. ChIP SDS lysis and dilution buffers were prepared according to Siddappa [43]. Chromatin was immunoprecipitated by overnight incubation at 4 °C with 1 μg of YAP1 antibody [44] or no antibody as a background control, followed by incubation 2 h at 4 °C with magnetic DynabeadsTM (ThermoFisher Scientific). Washing of the immunoprecipitate was done as previously described [43]. DNA purification using the Monarch PCR and DNA cleanup kit (New Englands BioLabs) was preceded by reversal of DNA–protein cross-links at 65 °C overnight, and by RNase and proteinase K treatments for 30 min and 2 h, respectively.

### Real-time PCR analyses

Total RNA from GCs was extracted, quantified and reverse transcribed as previously described [23]. PCR reactions consisted of 2.3 µl of water, 6 pmol of each forward and reverse gene specific primer (primer sequences are listed in Table S1 [45]) and 7.5 µl of Advanced qPCR mastermix with Supergreen Lo-ROX (Wisent Inc.) with 4 µl of reverse transcribed cDNA diluted tenfold. Relative mRNA levels were determined using CFX ManagerTM Software 3.0 (Bio-Rad), with the mathematical model according to Pfaffl [46] and using *Rpl19* as the housekeeping gene. Following ChIP, PCR reactions consisted 0.64 µl of water, 3.6 pmol of each forward and reverse primer and 9.64 µl of Advanced qPCR mastermix with 1 µl of purified chromatin. ChIP-qPCR assays were quantified with a standard curve derived from total DNA (input) and expressed as a percentage of input. The thermal cycling program was the same as previously described [23]. Real-time PCR reactions were run using a CFX96 Touch Real-Time PCR Detection System (Bio-Rad).

### Statistical analyses

Statistical analyses were done using GraphPad Prism software version 6.01 (GraphPad Software). Data are presented as means ± SEM. *P* ≤ 0.05 was considered statistically significant. Western blotting data were normalized by the sum of all data points in a replicate as previously described [47] and were subjected to arcsine transformation, as they are expressed as a proportion (0–1) of the whole replicate. Effects of gonadotropins on Hippo pathway effectors and target genes were analyzed by one-way ANOVA. GC cultures using pharmacologic inhibitors or adenoviruses were analyzed by two-way ANOVA. Dunnet’s and Tukey’s multiple comparison tests were used to identify differences with the control or between groups, respectively. Results of the ChIP-qPCR experiment were analyzed by two-way ANOVA followed by Sidak’s post hoc test.

## Results

### Hippo is activated by LH in vivo

**Fig. 1:**
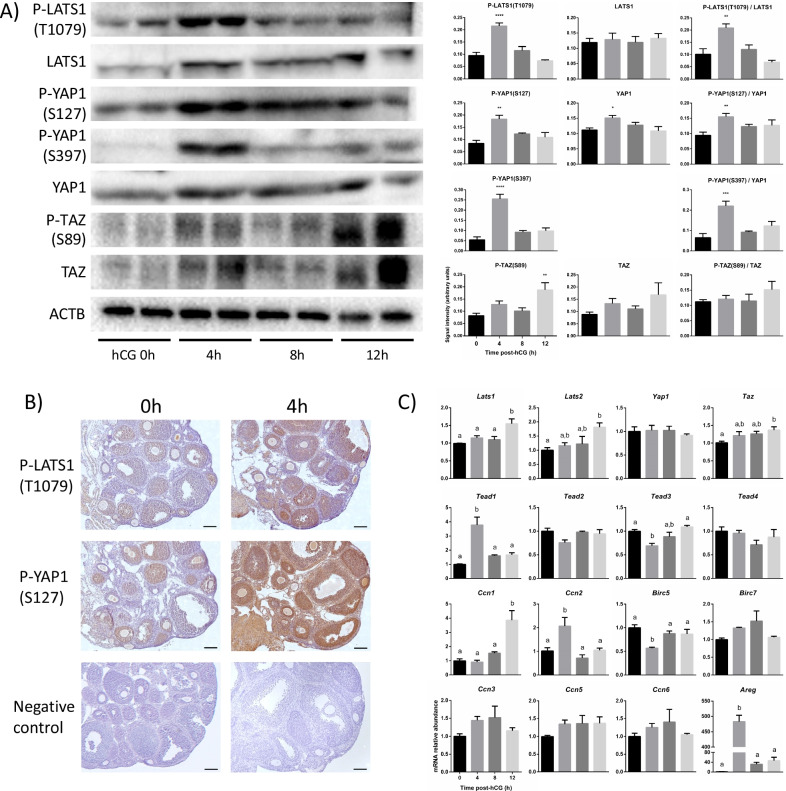
The ovulatory signal activates the Hippo pathway in granulosa cells in vivo. **A** Levels of Hippo pathway effectors in GCs of eCG-primed mice treated with hCG on a time course. Immunoblots show 2 replicates per time point, whereas quantification was done using 4 replicates per time point. Each replicate represents the ovaries of one mouse. β-actin (ACTB) was used as the loading control. Data were normalized by the sum of all data points in a replicate and were subjected to arcsine transformation, as they are expressed as a proportion (0–1) of the whole replicate. Data were compared to 0 h hCG. One-way ANOVA (Dunnet’s post hoc test): *P ≤ 0.05, **P ≤ 0.01, ***P ≤ 0.001, ****P ≤ 0.0001. **B** Immunohistochemical analyses of P-LATS1(T1079) and P-YAP1(S127) were performed on ovarian sections from immature eCG-primed mice prior to or after 4 h of hCG. Negative controls were incubated without the primary antibody. Scale bars are 100 μm. **C** RT-qPCR analysis of Hippo pathway effectors and target genes was performed on GCs isolated from immature eCG-primed mice treated with hCG on a time course (n = 4 mice/time point, done three times). All data were normalized to the housekeeping gene *Rpl19*. Different letters above histograms indicate significant differences between groups. One-way ANOVA (Tukey’s post hoc test): *P* ≤ 0.05

To investigate the effect of LH on Hippo signaling, GCs from immature (22- to 25-day old) eCG-primed WT mice were isolated 0, 4, 8 and 12 h following human chorionic gonadotropin (hCG) stimulation. Immunoblotting analyses showed that absolute and relative phosphorylation levels of LATS1 and of YAP1 were significantly increased at 4 h post-hCG (Fig. 1A). Phospho-TAZ levels were increased 12 h after treatment, but total TAZ levels tended to increase as well, resulting in an unchanged relative phosphorylation level (Fig. 1A). LATS2 protein levels were beneath the detection threshold (not shown). Immunohistochemical analyses showed that, prior to hCG stimulation (indicated as hCG 0 h), p-LATS1 and p-YAP1(Ser127) signals were faint and mainly localized to the GCs of growing follicles (Fig. 1B). A 4-h hCG treatment resulted in a readily apparent increase in p-LATS1 and p-YAP1 levels in GCs, mirroring the immunoblotting data. Levels of p-YAP1 were also elevated in the ovarian stroma after hCG stimulation (Fig. 1B). The effect of the ovulatory signal on the mRNA levels of Hippo pathway effectors and target genes was then determined in the GCs from hCG-treated mice. RT-qPCR data showed that hCG induced modest increases in *Lats1*, *Lats2* and *Taz* mRNA levels 12 h post-treatment, whereas *Yap1* transcript levels were not affected (Fig. 1C). Treatment with hCG also affected the expression of several canonical Hippo target genes belonging to families of the CCN ECM-associated proteins, BIRC apoptosis inhibitors and TEAD transcription factors. *Tead3* and *Birc5* mRNA levels decreased transiently 4 h post-hCG, whereas *Tead1* and *Ccn2* expression significantly increased. Interestingly, *Ccn1* expression increased 12 h after treatment. As expected, the expression of *Areg*, a major component of the LH signalling cascade and a known Hippo transcriptional target gene, increased dramatically 4 h after hCG stimulation (Fig. 1C). Taken together, these results demonstrate that the ovulatory signal activates the Hippo pathway as early as 4 h after hCG treatment, but that this activation does not result in a decrease either in YAP1/TAZ protein levels or in the mRNA levels of most YAP1/TAZ transcriptional target genes.

### In vitro stimulation of murine granulosa cells with LH activates the Hippo pathway

**Fig. 2:**
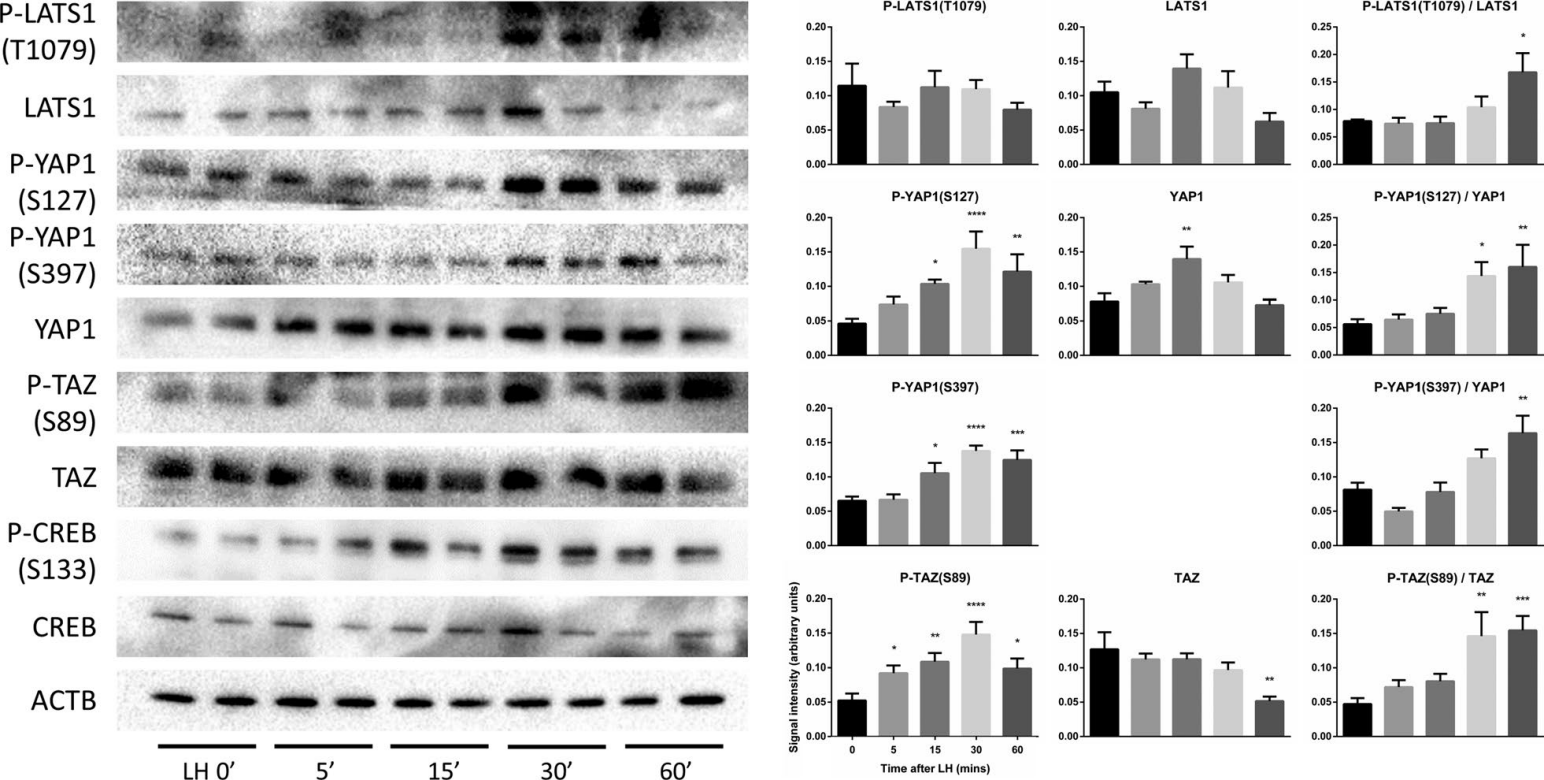
LH activates the Hippo pathway in granulosa cells in vitro. Primary GCs were treated with or without LH on a time course and the expression of the indicated proteins was evaluated by western blotting. Representative immunoblots show 2 replicates per time point, whereas quantification was done using 4 replicates per time point. β-actin (ACTB) was used as the loading control. Data were normalized by the sum of all data points in a replicate and were subjected to arcsine transformation, as they are expressed as a proportion (0–1) of the whole replicate. Data were compared to 0 min LH. One-way ANOVA (Dunnet’s post hoc test): *P ≤ 0.05, **P ≤ 0.01, ***P ≤ 0.001, ****P ≤ 0.0001

The regulation of Hippo signaling by LH was also investigated in vitro. GCs were isolated from eCG-primed immature mice, placed in culture and treated with human recombinant LH on a time course. As observed in vivo, treatment resulted in the activation of the Hippo pathway. Increased relative levels of p-LATS1(Thr1079), p-YAP1(Ser127 and Ser397) and p-TAZ(Ser89) were observed in response to LH, with peak levels being observed as soon as 30 min after treatment (Fig. 2). LH stimulation had only minimal effects on total protein levels. CREB phosphorylation was used as a positive control to validate the effectiveness of the LH treatment and increased rapidly in response to treatment as expected. These results demonstrate that in vitro treatment of murine GCs with LH induces a rapid activation of the Hippo pathway similar to what is observed in vivo, further supporting our hypothesis that Hippo mediates the LH response.

### PKA mediates the activation of the Hippo pathway by LH

**Fig. 3:**
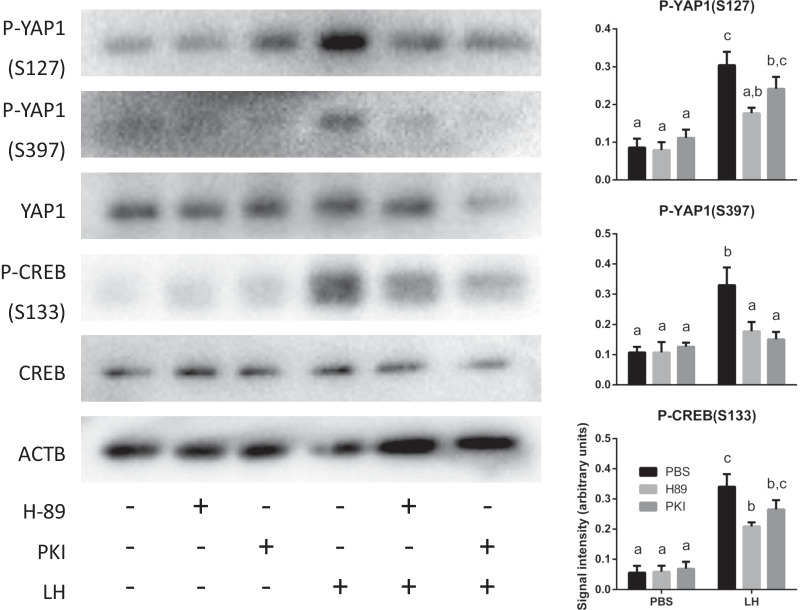
LH acts via PKA to phosphorylate YAP1. Primary cultured GCs were pretreated with or without inhibitors against PKA (H-89 or PKI) followed by treatment with LH or vehicle control for 30 min. Representative immunoblots show 1 replicate per condition, whereas quantification was done using 4 replicates per condition. β-actin (ACTB) was used as the loading control. Data were normalized by the sum of all data points in a replicate and were subjected to arcsine transformation, as they are expressed as a proportion (0–1) of the whole replicate. Different letters above histograms indicate significant differences between treatment conditions. Two-way ANOVA (Tukey’s post hoc test): *P* ≤ 0.05

To determine the mechanism whereby LH activates Hippo, the three major signaling pathways activated downstream of the LH receptor were evaluated, namely cAMP/PKA, PI3K/AKT and ERK1/2 pathways. Cultured eCG-primed WT GCs were pretreated (or not) with the PKA inhibitors H-89 and PKI, the AKT1/2/3 inhibitor MK-2206 or the MEK1/2 inhibitor U0126, followed by treatment with LH for 30 min. Treatment with inhibitors had no effect on basal levels of YAP1 phosphorylation (at Ser127 or Ser397; Fig. 3). However, LH-dependent phosphorylation of YAP1 on Ser127 and Ser397 was inhibited by H-89 and PKI (Fig. 3, S1 [48] and S2 [49]). MK-2206 or U0126 alone (Fig. S1 [48]) or combined (Fig. S2 [49]) had no effect on LH-induced YAP1 phosphorylation. Likewise, their concomitant pretreatment with H-89 did not result in a greater effect than H-89 alone (Fig. S2 [49]). All inhibitors were effective, as they were able to inhibit LH-dependent phosphorylation of CREB (H-89, PKI), AKT (MK-2206) and ERK1/2 (U0126) (Fig. 3, S1 [48] and S2 [49]). Together, these results indicate that LH acts via the cAMP/PKA pathway to activate Hippo signaling in murine GCs.

### *Yap1*/*Taz* knockdown blunts the LH response by preventing ERK1/2 activation

**Fig. 4:**
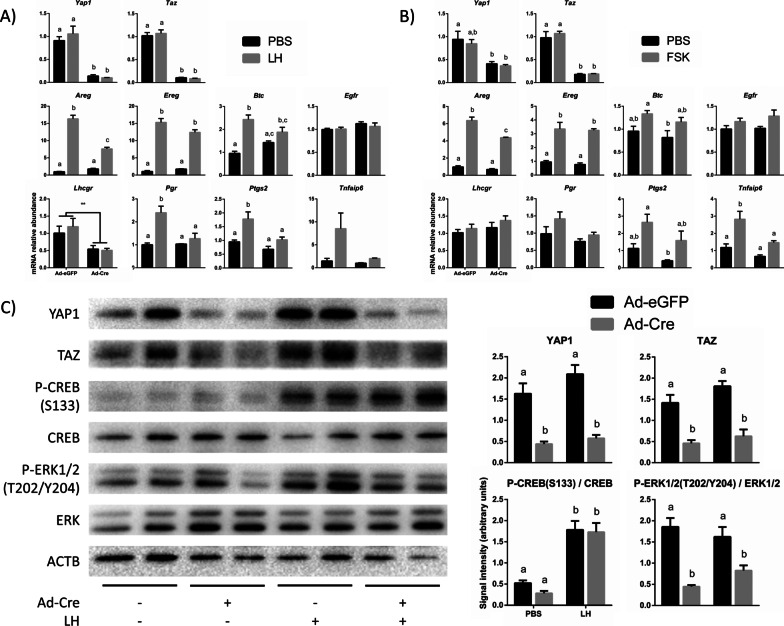
Loss of *Yap1* and *Taz* blunts LH responsiveness. **A**, **B** Primary GCs isolated from *Yap1*^f/f^;*Taz*^f/f^ mouse ovaries were infected with adenoviruses expressing either Cre recombinase (Ad-Cre; to knockdown *Yap1* and *Taz* expression) or eGFP (Ad-eGFP; control) for 18 h. GCs were treated with either LH (**A**), FSK (**B**) or PBS (control) for 2 h (n = 4 replicates/treatment, done three times). Data were normalized to the housekeeping gene *Rpl19*. C) Primary *Yap1*^f/f^;*Taz*^f/f^ GCs were infected with Ad-Cre for 24 h (Ad-eGFP for control). GCs were treated with LH (PBS for control) for 30 min. Representative immunoblots show 2 replicates per condition, whereas quantification was done using 12 replicates per condition. β-actin (ACTB) was used as the loading control. Data were all normalized by the sum of all data points in a replicate and were subjected to arcsine transformation, as they are expressed as a proportion (0–1) of the whole replicate. Different letters above histograms indicate significant differences between treatment conditions. Two-way ANOVA (Tukey’s post hoc test): *P* ≤ 0.05

After elucidating the mechanism by which Hippo is activated downstream of the LH receptor (LHCGR), the potential role of Hippo signaling in mediating LH action was investigated. To do so, GCs isolated from mice bearing floxed alleles for *Yap1* and *Taz* (*Yap1*^f/f^;*Taz*^f/f^) were placed in culture and infected with adenoviruses to drive expression of eGFP (Ad-eGFP, control) or Cre recombinase (Ad-Cre, to inactivate the floxed alleles), followed or not by treatment with LH for 2 h. In this model, knockdown of *Yap1* and *Taz* mRNA levels of approximately tenfold were achieved after 18 h of adenovirus treatment (Fig. 4A). LH-induced expression of *Areg*, *Pgr* and *Ptgs2*—but not *Ereg*, *Btc* and *Egfr*—was significantly impaired by *Yap1*/*Taz* knockdown (Fig. 4A). Similar results were obtained with 1 and 6 h of LH treatment (Fig. S3 [50]). Among the genes investigated, *Lhcgr* was the only gene whose expression was downregulated following loss of *Yap1*/*Taz*, regardless of LH treatment. To determine if the above-mentioned blunted induction of LH target genes was simply due to *Lhcgr* downregulation, a similar experiment was conducted using forskolin (FSK—a stimulator of adenylate cyclase, which acts downstream of the LHCGR to produce cAMP). This showed that the FSK-mediated induction of *Areg*, *Ptgs2* and *Tnfaip6* was blunted by the loss of *Yap1*/*Taz* similarly to the LH-treated cells (Fig. 4B). This suggests that YAP1/TAZ participate in the transduction of the LH signal somewhere downstream of cAMP.

To identify the components of the LH cascade affected by the loss of *Yap1*/*Taz*, we conducted a similar experiment and evaluated phosphorylation levels of CREB and ERK1/2. Ad-Cre treatment for 24 h lowered YAP1/TAZ protein levels to approximately 30–40% those of the controls (Fig. 4C). As expected, treatment with LH activated the cAMP/PKA and ERK1/2 pathways. Loss of YAP1/TAZ did not affect phosphorylation levels of CREB on its activating site (Ser 133), suggesting that the cAMP/PKA/CREB component of the LH signalling cascade remained intact (Fig. 4C). However, loss of YAP1/TAZ resulted in decreased phosphorylation of the ERK1/2 kinases on threonine 202 and tyrosine 204 residues (Fig. 4C), both with or without LH stimulation. Taken together, these results indicate that YAP1/TAZ are necessary for the adequate activation of the ERK1/2 pathway—but not the PKA/CREB pathway—and for the induction of several LH target genes in murine GCs.

### *Yap1* and *Taz* regulate a subset of LH target genes

**Fig. 5:**
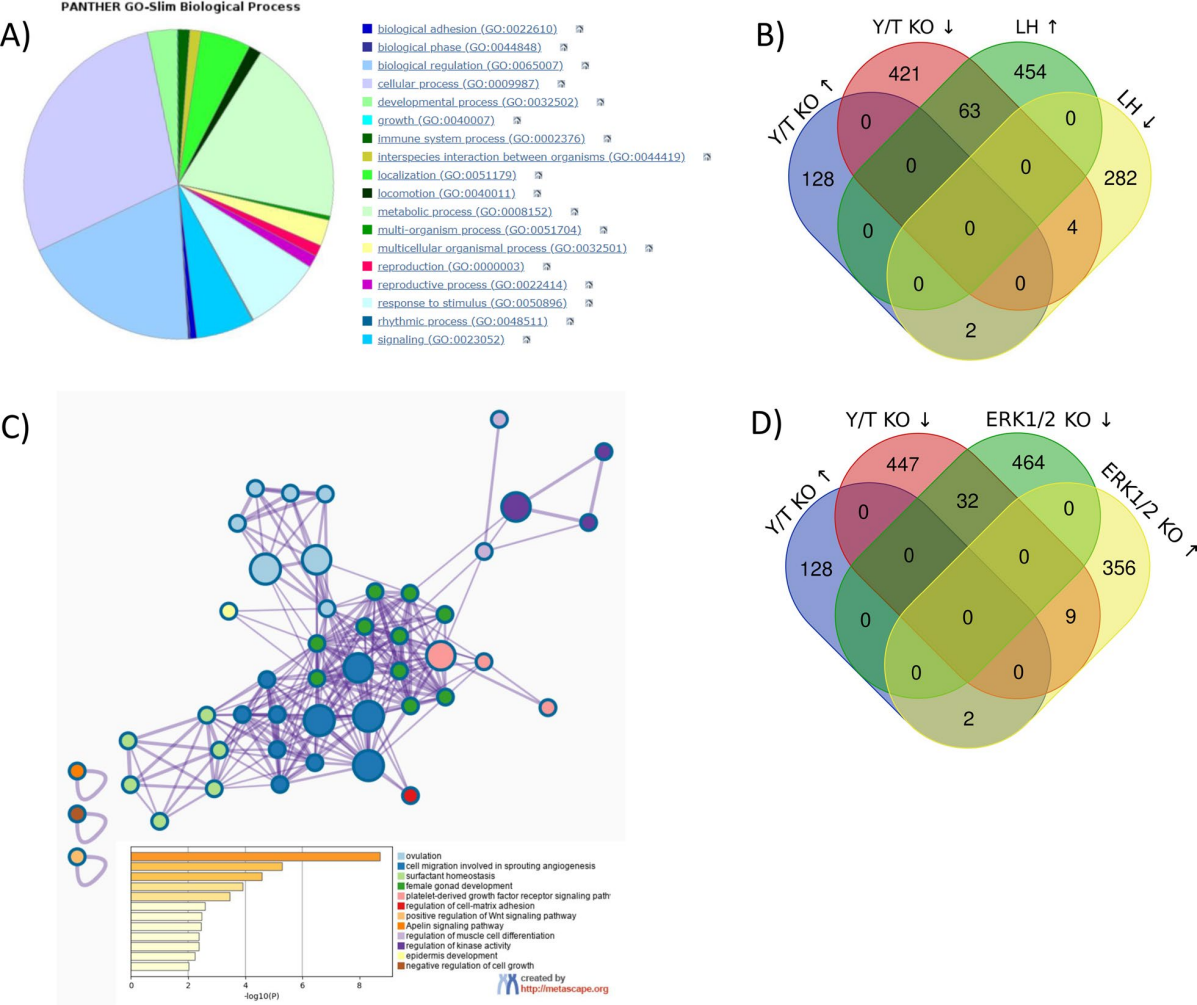
Loss of *Yap1*/*Taz* expression in murine granulosa cells negatively affects the expression of several ovulation-related genes. Granulosa cells isolated from *Yap1*^f/f^;*Taz*^f/f^ mice were infected with adenoviruses expressing either Cre recombinase (Ad-Cre; to knockdown *Yap1* and *Taz* expression) or eGFP (Ad-eGFP; control) for 18 h. A microarray analysis was conducted to identify differentially expressed genes (DEGs). **A** Pie chart representing the Panther gene-ontology (GO) functional classification of the DEGs following *Yap1* and *Taz* knockdown. **B** Venn Diagram highlighting the overlap between DEGs following loss of *Yap1*/*Taz* expression *vs* LH stimulation for 3 h in primary murine GCs. **C** Metascape enrichment network visualization showing clustering of the Hippo and LH-dependent genes according to their GO biological processes. Cluster annotations are shown in color code next to the bar graph representing statistical significance (only *P* < 0.01 are shown) of the said enriched biological processes. **D** Venn Diagram highlighting the overlap between DEGs following loss of *Yap1*/*Taz* expression *vs Erk1*/*2*-deficient GCs treated with hCG for 2.5 h

In order to highlight specific roles of YAP1/TAZ in murine GC physiology, we conducted a microarray analysis of genes differentially expressed in cultured *Yap1*^f/f^;*Taz*^f/f^ GCs infected for 18 h with the Ad-Cre virus or Ad-eGFP (control). We found that 130 genes were upregulated and 488 were downregulated in cells in which *Yap1*/*Taz* were knocked down. Using the PANTHER Classification System, we were able to show that these genes are involved in various biological processes, a small proportion of which related to reproduction (red and magenta—Fig. 5A). We then compared this data with a microarray analysis of differentially expressed genes following LH treatment of GCs isolated from WT mice. We show that a sizeable proportion of genes downregulated in the absence of *Yap1*/*Taz* (≈13%, 63 of 488) are the same genes that are induced by LH (Fig. 5B, Table 1). Analysis of this sub-fraction of genes using the Metascape Anotation tool highlighted biological processes related to ovulation, angiogenesis and female gonad development (blue and dark green—Fig. 5C, Table 1). Additional analysis with DAVID identified similar biological processes (Table S2 [51]). Given that *Yap1*/*Taz* knockdown prevented the adequate activation of the ERK1/2 pathway (Fig. 4C), we then compared our analysis of genes differentially expressed following *Yap1*/*Taz* knockdown to previously published microarray data of *Erk*1/2-depleted GCs from eCG-primed mice 2.5 h after hCG stimulation [4]. Among all the genes downregulated following *Yap1*/*Taz* knockdown, 32 of them were also downregulated following *Erk*1/2 loss (Fig. 5D, Table 1). As expected, bioinformatic analysis of this subfraction of genes using Metascape and DAVID software identified biological processes of ovulation, angiogenesis and transmembrane receptor protein tyrosine kinase signaling pathway (Table S3 [52]). The above results therefore indicate that YAP1/TAZ are involved in the regulation of several LH- and ERK1/2-dependent genes, but also regulate large numbers of additional genes in murine GCs.

**Table 1:** List of genes downregulated following loss of *Yap1*/*Taz* whose expression is LH and/or *Erk1*/*2*-dependent

Gene Symbol	FC in *Yap1*/*Taz* depleted GCs	FC in LH-stimulated GCs	FC in *Erk1*/*2* depleted GCs
Abi3bp	− 1.60		− 1.91
Arhgap32	− 1.51	1.62	
Arid5b	− 1.90		− 1.56
Arrdc3	− 1.88	2.94	
Bpgm	− 1.57	1.61	
Bzw1	− 1.72	1.51	
Cblb	− 1.59		− 2.46
Ccnl1	− 1.56	1.88	
Ccno	− 1.76	4.41	
Cited2	− 2.00		− 2.00
Cpne3	− 1.52		− 1.96
Efnb2	− 1.63	1.58	− 4.58
Egr1	− 1.62		− 37.71
Emb	− 1.53		− 3.67
Emp1	− 1.69		− 5.60
Ereg	− 1.55	7.70	− 2.09
Errfi1	− 2.25		− 6.00
Fam107b	− 1.95	1.77	
Far1	− 1.75	1.67	
Fbxw14	− 1.70	2.95	
Fbxw15	− 1.62	3.51	
Fbxw19	− 1.86	2.89	
Fbxw20	− 2.14	3.08	
Fbxw21	− 2.88	2.40	
Fbxw22	− 1.64	2.26	
Ggct	− 1.56	1.55	
Glrx	− 1.92	1.54	
Gng3	− 1.94	1.64	
Hccs	− 1.93		− 1.61
Hsd3b1	− 1.81	1.54	
Igf2bp2	− 1.83	1.69	
Il23a	− 1.59		− 2.67
Inhba	− 2.02	3.64	
Jag1	− 2.03	1.62	− 1.75
Kdr	− 1.94	1.69	
Khdc1a	− 1.63	1.69	
Lrrc8c	− 1.54		− 1.86
Mid1	− 1.58	1.87	− 3.12
Nfya	− 1.97	1.52	
Nlrp14	− 2.09	2.69	
Nlrp2	− 1.53	1.87	
Nlrp4a	− 2.31	1.74	
Oas1c	− 1.64	2.78	
Oas1e	− 2.15	2.80	
Osgin2	− 1.96	3.23	− 1.69
Phldb2	− 1.61		− 4.09
Pik3r1	− 1.73		− 2.63
Plat	− 1.80	1.56	
Plau	− 1.54	2.03	− 10.36
Plk2	− 1.59	3.71	− 2.47
Prkg2	− 1.59		− 2.64
Ptgs2	− 3.06	1.98	− 42.47
Ptp4a1	− 1.51	2.42	
Rbm47	− 1.96	1.54	− 3.11
Rgs2	− 2.13	3.05	
Rnd3	− 1.72	1.51	− 12.90
Rsbn1	− 1.78	1.76	
Rspo2	− 1.55	1.88	
Serpinb2	− 1.66		− 4.39
Sfrp4	− 1.99	1.93	− 6.40
Siah1b	− 1.54	1.64	
Slc7a8	− 2.36	2.09	− 1.69
Spin1	− 2.81	1.56	
Tcl1b1	− 1.93	1.91	
Tiparp	− 2.00	1.66	− 1.63
Tnfaip6	− 2.27	6.65	− 10.01
Zfp804a	− 1.68		− 3.50
Znrf2	− 1.63		− 2.03

Genes listed are downregulated following 18 h of *Yap1*/*Taz* knockdown in GCs, and meet one of the two following criteria: (1) upregulated in WT GCs stimulated 3 h with LH, or (2) downregulated in *Erk1*/*2*-deficient GCs isolated 2.5 h after hCG stimulation. Genes are listed in alphabetical order. Fold change (FC) is expressed as a ratio between the treatment (Ad-Cre, LH, *Erk1*/*2* depleted GCs) and its respective control (Ad-eGFP, PBS, WT GCs). Oocyte-specific genes were excluded from this list as their presence was likely caused by oocyte contamination of our primary GC culture. Cut-off thresholds of *P* ≤ 0.05 and |FC|≥ 1.5 were used to identify differentially expressed genes

### YAP1 regulates LH responsiveness through the direct transcriptional regulation of *Areg*

**Fig. 6:**
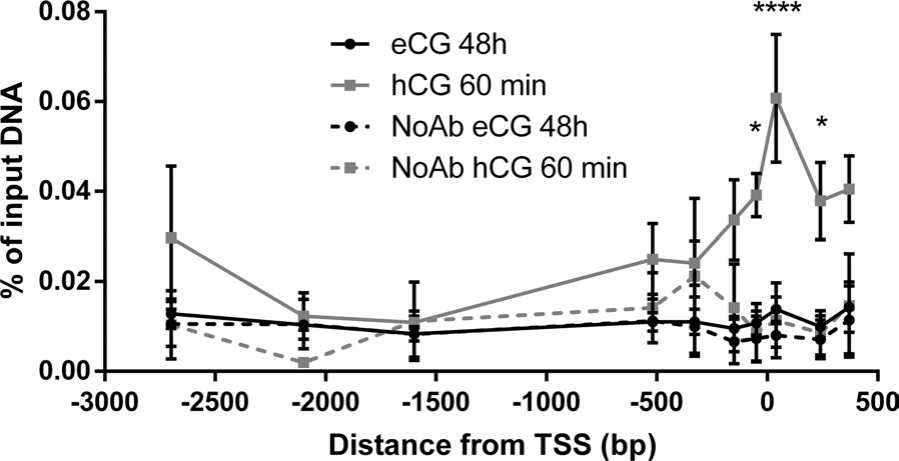
YAP1 is recruited to the *Areg* promoter following hCG stimulation. ChIP-qPCR analyses were performed on GCs isolated from eCG-primed WT mice before (black lines) or after (grey lines) 60 min of hCG stimulation to evaluate the enrichment of YAP1 on the *Areg* promoter. The no antibody controls (NoAb) appear as dotted lines. n = 4 biological replicates/timepoint, one replicate contains the GCs of 4 mice. Data are expressed as percentage of input DNA. Two-way ANOVA (Sidak’s post hoc test): *P ≤ 0.05, ****P ≤ 0.0001

We showed that loss of *Yap1*/*Taz* results in a blunted LH-dependent induction of *Areg* and in a decreased activation of the ERK1/2 pathway (Fig. 4). Given that AREG activates ERK1/2 via the EGF receptor, and *Areg* has been previously shown to be a direct transcriptional target of the Hippo pathway in other cellular contexts [19, 20], we hypothesized that YAP1 regulates LH responsiveness in GCs through the transcriptional regulation of *Areg*. A chromatin immunoprecipitation (ChIP) experiment was therefore conducted using a YAP1 antibody on GCs isolated from eCG-primed mice treated (or not) with hCG for 60 min, followed by an in silico analysis of the *Areg* promoter region (from − 4500 to + 1300 bp around the TSS) to locate DNA motifs of the YAP1/TAZ binding partners belonging to the TEAD family of transcription factors (TEAD4; ≈ − 2150 and − 2950 from the *Areg* transcription start site [TSS]) (Additional file 1 [53]). qPCR analyses of chromatin fragments enriched from the ChIP were performed using primers designed to amplify regions surrounding the aforementioned motifs and other regions closer to the TSS. Unexpectedly, there was no enrichment of YAP1-bound DNA fragments around the TEAD4 motifs. Instead, YAP1 was significantly (≈fivefold) recruited near the *Areg* TSS 60 min following hCG treatment (Fig. 6). In contrast, GCs isolated from mice that were only primed with eCG (that did not receive hCG) had low YAP1-enrichment levels, comparable to the no-antibody control (Fig. 6). These results indicate that YAP1 is rapidly recruited to the promoter of *Areg* following hCG stimulation.

## Discussion

### Amphiregulin is transcriptionally regulated by Hippo in murine granulosa cells

**Fig. 7:**
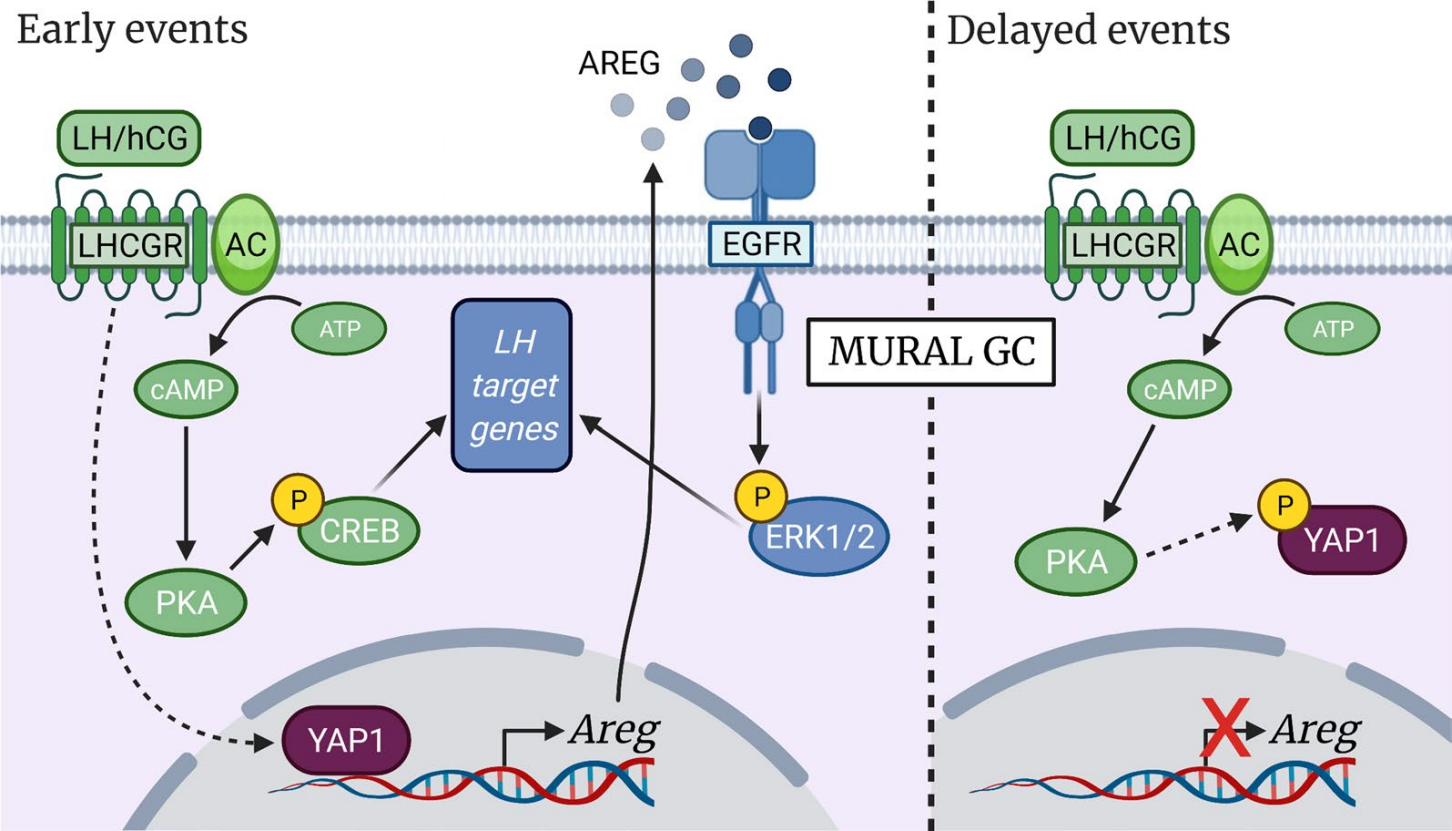
The Hippo pathway plays an essential role in the regulation of the LH cascade. **Early events**: Following binding of LH to its receptor, CREB is activated and YAP1 is recruited in proximity to the transcription start site of the *Areg* promoter. The latter contributes to the rapid upregulation of *Areg* in response to the LH stimulus. High extracellular levels of AREG contribute to the activation of the EGFR and downstream ERK1/2 pathway, and to the regulation of several LH-dependent genes. **Delayed events**: Granulosa cell stimulation by LH subsequently activates the Hippo pathway, resulting in YAP1 phosphorylation through a PKA-dependent mechanism. This Hippo activation is thought to be necessary for the transient expression pattern of *Areg* and for prolonged LH effects. Dashed arrows indicate a relation for which unidentified intermediate effectors may be involved. Created with BioRender.com

Successful ovulation is dependent on the orchestrated response of every follicular cell type to the LH surge. *Areg* has a central role in the transmission of the LH signal to cumulus cells and oocyte, neither of which express *Lhcgr*. Its rapid upregulation and release in the extracellular space of mural granulosa cells in response to the LH signal is necessary for the activation of EGFR and, subsequently, ERK1/2 (Fig. 7). This in turn leads to cumulus expansion, oocyte maturation, luteinization and follicle wall rupture. YAP1 has been shown to directly regulate *Areg* transcription in a mammary gland epithelial cell line [19], although the transcription factor to which YAP1 binds in this context has not been identified. More recently, TAZ has been reported to directly regulate *AREG* expression via interaction with TEAD and subsequent binding to a TEAD binding site 1.8 kb upstream of the *AREG* TSS [20]. In the present study, we found a marked recruitment of YAP1 at the proximal promoter of *Areg* in GCs isolated from WT mice 60 min after hCG stimulation (Fig. 7). YAP1 enrichment was located near the TSS and not around putative TEAD4 binding sites in the *Areg* promoter. It is possible that YAP1 interacts with a yet unidentified transcription factor that bind near the TSS. YAP1 recruitment near the *Areg* TSS might also be the result of an interaction of the basal transcriptional machinery with YAP1 bound to an unidentified distal enhancer, perhaps located beyond the region that was investigated by ChIP-qPCR. Such interaction between YAP1/TAZ/TEAD-bound enhancers with promoters via chromatin looping has been reported in the *MYC* and *TOP2A* genes in an epithelial human breast cancer cell line [54]. In our model, we were unable to identify the transcription factor(s) that YAP1 interacts with to regulate the transcription of *Areg*, nor the precise location of their relevant DNA binding site(s). Nevertheless, our results demonstrate for the first time that *Areg* is a direct transcriptional target of Hippo in the early events following the ovulatory signal in murine GCs.

### YAP1/TAZ are required for the adequate induction of several LH target genes and for the activation of the ERK1/2 pathway

In this study, we demonstrate the role of YAP1/TAZ in the induction of several ovulation-related genes following LH stimulation. Only one other previous study investigated the role of *Yap1* in regulating LH transcriptional target genes in murine GCs. It showed that *Yap1* knockdown prior to GC stimulation with forskolin (FSK) and phorbol 12-myristate 13-acetate (PMA) resulted in increased levels of *Areg*, *Ereg*, *Btc*, *Ptx3*, *Sult1e1* and *Lhcgr* expression [17]. The same study also reported lower induction levels of *Areg*, *Ereg*, *Sult1e1* and *Tnfaip6* by FSK + PMA following 24 h of YAP1 overexpression. These findings therefore appear to contradict those reported in this study. The discrepancies could be explained by differences in cell culture conditions, methods used to manipulate *Yap1*/*Taz* expression, the nature of the agonists that were used and the duration of treatments. Pretreatment of bovine mural GCs with verteporfin (VP), an inhibitor of YAP1-TEAD interaction [55], was shown to prevent the upregulation of *Ereg* by LH [56]. This difference might be explained by a greater role of *Ereg* (and a lesser role for *Areg*) in the acute response of bovine mural GCs to LH [57].

The present study also identified lower basal levels of ERK1/2 activity following *Yap1*/*Taz* knockdown, highlighting the requirement for *Yap1*/*Taz* expression in GCs even prior to LH stimulation. A similar effect on ERK1/2 activity was previously shown in bovine GCs treated with inhibitors of YAP1-TEAD interaction [56]. As ERK1/2 regulate the expression of a wide variety of genes involved in the ovulation process [4], this lower basal level of ERK1/2 activity is purported to be the cause of the blunted induction of ovulation-related genes, which was observed *Yap1*/*Taz*-deficient GCs in response to LH. The EGFR pathway was recently identified as a likely mediator of YAP1 actions in murine GCs, as *Yap1* knockdown dramatically reduced *Egfr* expression [16]. Differences in the knockdown method used might explain why *Egfr* expression was unchanged in our *Yap1*/*Taz* knockdown model. Importantly, loss of *Yap1*/*Taz* expression had no effect on PKA/CREB pathway activation by LH. For this reason, the effects of YAP1/TAZ disruption could not be attributable solely to *Lhcgr* downregulation, as PKA/CREB activity would also have been affected. This further points to AREG as a likely mediator of YAP1/TAZ regulation of LH signaling, as it binds to EGFR to activate ERK1/2 signaling without affecting PKA. Together, these findings indicate that YAP1/TAZ activity is required prior to the LH surge to allow the adequate induction of several LH-dependent genes. The mechanism of YAP1/TAZ action in this context involves the regulation of the ERK1/2 pathway and is independent of PKA/CREB activation (Fig. 7).

### PKA-dependent Hippo activation following the LH surge

Inactivation of the Hippo pathway promotes nuclear localization of YAP1 and TAZ, their interaction with transcription factors and subsequent transcriptional co-regulatory activity. It is well described in the literature that Hippo inactivation is necessary for GC proliferation and steroidogenesis [14, 15]. The activation of the Hippo pathway reported in the current study is in line with results of previous reports that showed YAP1 and TAZ phosphorylation following the ovulatory signal [16–18]. In addition, this study shows that LH promotes phosphorylation of LATS1. Using pharmacologic inhibitors, we showed that the mechanism responsible for Hippo activation by LH involves PKA (Fig. 7) and is independent of the PI3K/AKT and ERK1/2 pathways. This finding is consistent with a previous report of phosphorylation of YAP1 by a constitutively active PKA in rat GCs [58]. The cAMP/PKA pathway has also been reported to be involved in YAP1 phosphorylation in other cellular contexts [59–61]. Ji et al. [17] identified the ERK1/2 pathway as likely being involved in Hippo activation following the LH signal. Using *Erk1*/*2* depleted GCs and the same MEK1/2 inhibitor used in the present study, they concluded that an intact ERK1/2 pathway is needed for the phosphorylation of YAP1. However, in our model, pharmacologic inhibition of the ERK1/2 pathway had no effect on YAP1 phosphorylation levels. Again, differences in experimental conditions including culture methods and timing may explain these discrepancies.

Although our results showing that phosphorylation/inactivation of YAP1/TAZ occurs in response to the ovulatory signal might appear to conflict with the aforementioned requirement of YAP1/TAZ for the induction LH-dependent genes, we believe that these two findings are, in fact, compatible. Prior to (and likely during the early steps of) activation of the LH cascade, YAP1/TAZ transcriptional co-regulatory activity appears necessary. However, their nuclear presence may subsequently inhibit the longer-term effects of LH, thus necessitating Hippo activation to abrogate their transcriptional activity. Indeed, YAP1 overexpression in GCs has been shown to impair progesterone production, a marker of luteal cell activity [16]. Further experiments involving the sustained activation of YAP1 and TAZ in the hours following hCG stimulation may confirm their negative impact on LH action in the longer term. It should be noted that the induction of *Areg* by LH is both rapid and transient [5] (Fig. 1C), suggesting that, while YAP1 participates in the initial increase in its transcription, Hippo activation could then contribute to its subsequent downregulation by phosphorylating YAP1.

### YAP1/TAZ regulates a wide variety of genes in granulosa cells, many of which are involved in biological processes related to ovulation

Another important finding reported in the present study is the large proportion of genes downregulated following *Yap1*/*Taz* knockdown that are upregulated by LH in murine GCs. Among these overlapping genes, many play well-established roles in the ovulation process, such as *Ereg*, *Hsd3b1*, *Inhba*, *Kdr*, *Plat*, *Plau*, *Plk2*, *Ptgs2*, *Sfrp4* and *Tnfaip6*. Genes involved in angiogenesis were also overrepresented, which was expected considering that the growth of blood vessels is a hallmark in the formation of the corpus luteum [62]. Although others have previously identified and categorized differentially expressed genes in GCs undergoing luteinization [63], our finding that YAP1/TAZ are required for the adequate expression of a large set of ovulation-related genes in this context is novel. Nonetheless, our microarray results also indicate that most genes differentially expressed following YAP1/TAZ knockdown are unrelated to LH actions. This suggests a wider involvement of the Hippo pathway in GC physiology that will require further investigation.

## Conclusions

In summary, the present study elucidated mechanisms involved in the interplay between the Hippo pathway and the LH cascade (Fig. 7). YAP1/TAZ were found to be required for the induction of a variety of LH-dependent genes, likely through the activation of the ERK1/2 pathway. The LH signalling cascade was also found to activate Hippo through a PKA-dependent mechanism. This study also reports for the first time the direct transcriptional regulation of *Areg* by YAP1 in GCs, providing a plausible mechanism whereby YAP1 may mediate the activation of ERK1/2. Further investigation into the interactions between the Hippo pathway and the LH cascade in granulosa cells would benefit our understanding of the ovulation process and its many components. This will in turn allow the development of more targeted therapies for the treatment of ovarian diseases such as polycystic ovarian syndrome, the inhibition of ovulation for contraceptive purposes, or the optimization of ovarian stimulation protocols crucial to IVF.

## Supplementary Information

**Additional file 1.** In silico analysis of the Areg promoter region to locate DNA binding motifs.

## Data Availability

All data generated or analysed in this study are either included in this published article or deposited in the GEO database under Accession No. GSE184396.

## Abbreviations

Ad-Cre: Cre recombinase adenovirus
Ad-eGFP: EGFP control adenovirus
Areg: Amphiregulin
Birc5-7: Baculoviral inhibitors of apoptosis repeat containing 5–7
Btc: Betacellulin
cAMP: Cyclic adenosine monophosphate
CCN1–2–3–5–6: CCN family member 1–2–3–5–6
CREB: CAMP-response element binding protein
DEGs: Differentially expressed genes
eCG: Equine chorionic gonadotropin
EGF: Epidermal growth factor
EGFR: Epidermal growth factor receptor
Ereg: Epiregulin
ERK1/2: Extracellular signal-regulated kinases 1 and 2
FSK: Forskolin
GC: Granulosa cell
hCG: Human chorionic gonadotropin
LATS1/2: Large tumor suppressor kinases 1 and 2
LH: Luteinizing hormone
Lhcgr: Luteinizing hormone/choriogonadotropin receptor
Pgr: Progesterone receptor
PKA: Protein kinase A
Ptgs2: Prostaglandin-endoperoxide synthase 2
RUNX: Runt-related
Ser: Serine
TAZ: Transcriptional coactivator with PDZ-binding motif
Tead1–2–3–4: TEA domain transcription factor 1–2–3–4
Tnfaip6: TNF apha induced protein 6
YAP1: Yes-associated protein 1
