# Supplementary material for: The granulosa cell response to luteinizing hormone is partly mediated by YAP1-dependent induction of amphiregulin
Source: Cell Commun Signal. 2022 May 26;20:72. doi: 10.1186/s12964-022-00843-1 (PMC9137176; doi:10.1186/s12964-022-00843-1)
Supplement: Supplementary file 2 — Additional file 1. In silico analysis of the Areg promoter region to locate DNA binding motifs. [file 12964_2022_843_MOESM2_ESM.pdf]

Raw images used to quantify WBs

The Granulosa Cell Response to Luteinizing Hormone is Partly Mediated by YAP1-Dependent Induction of Amphiregulin

By: Philippe Godin, Mayra F Tsoi, Martin Morin, Nicolas Gévry and Derek Boerboom

FIGURE 1

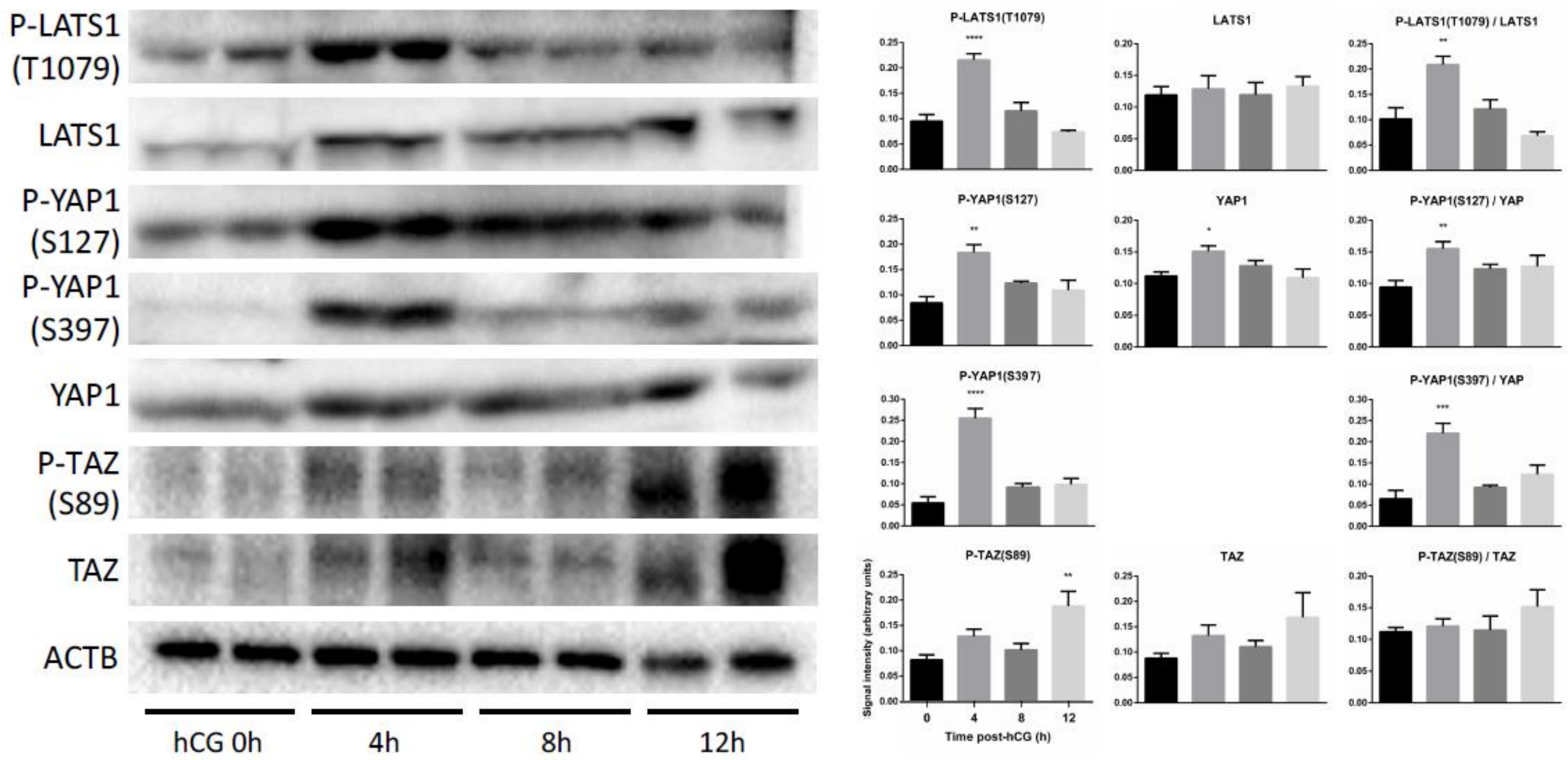
P-LATS1 (T1079)
LATS1
P-YAP1 (S127)
P-YAP1 (S397)
YAP1
P-TAZ (S89)
TAZ
ACTB
hCG 0h
4h
8h
12h
P-LATS1(T1079)
LATS1
P-LATS1(T1079) / LATS1
P-YAP1(S127)
YAP1
P-YAP1(S127) / YAP
P-YAP1(S397)
P-YAP1(S397) / YAP
P-TAZ(S89)
TAZ
P-TAZ(S89) / TAZ
Signal intensity (arbitrary units)
Time post-hCG (h)

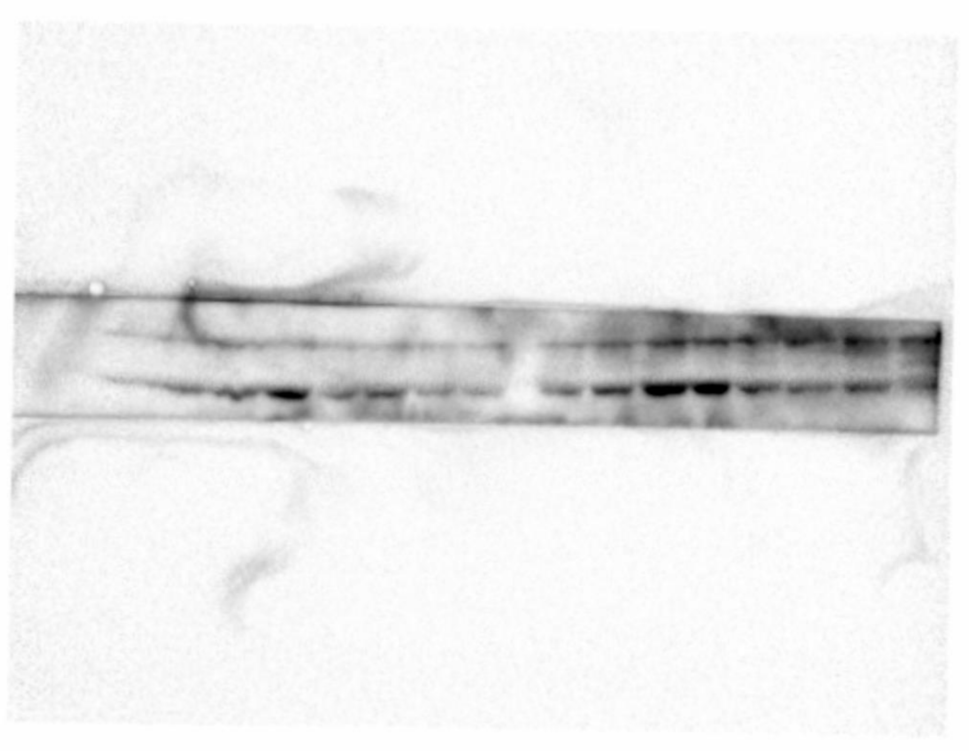

P-lats1

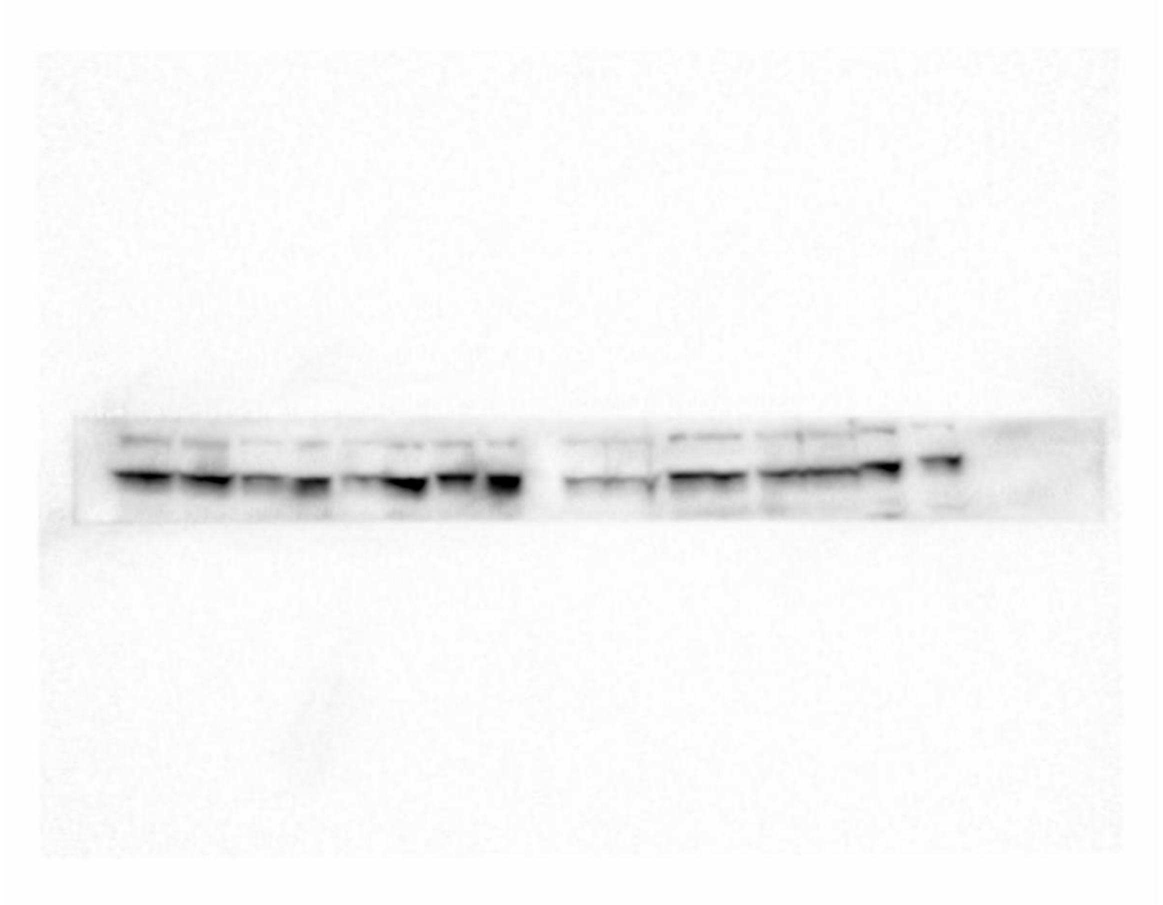

Lats1

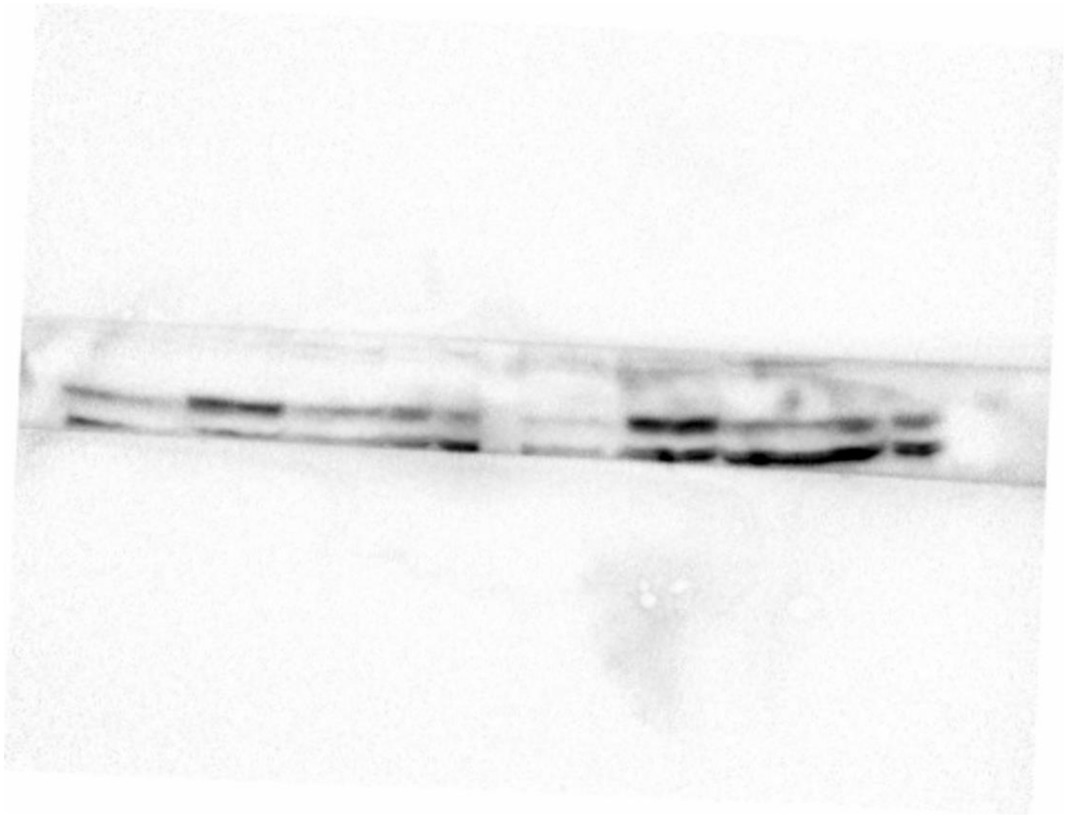

P-Yap1 397

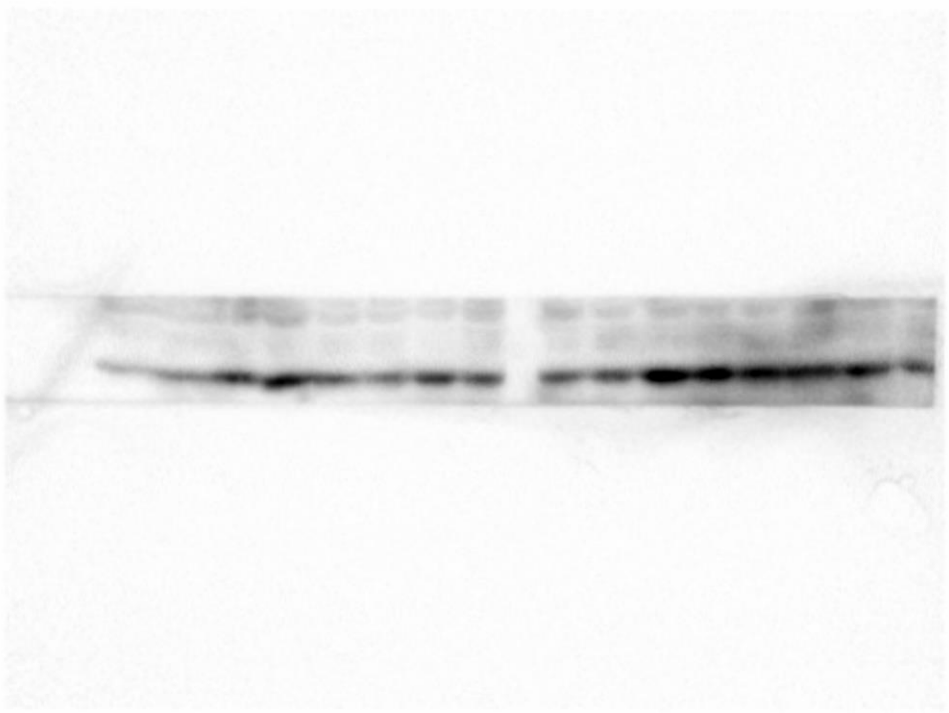

P-Yap1 127

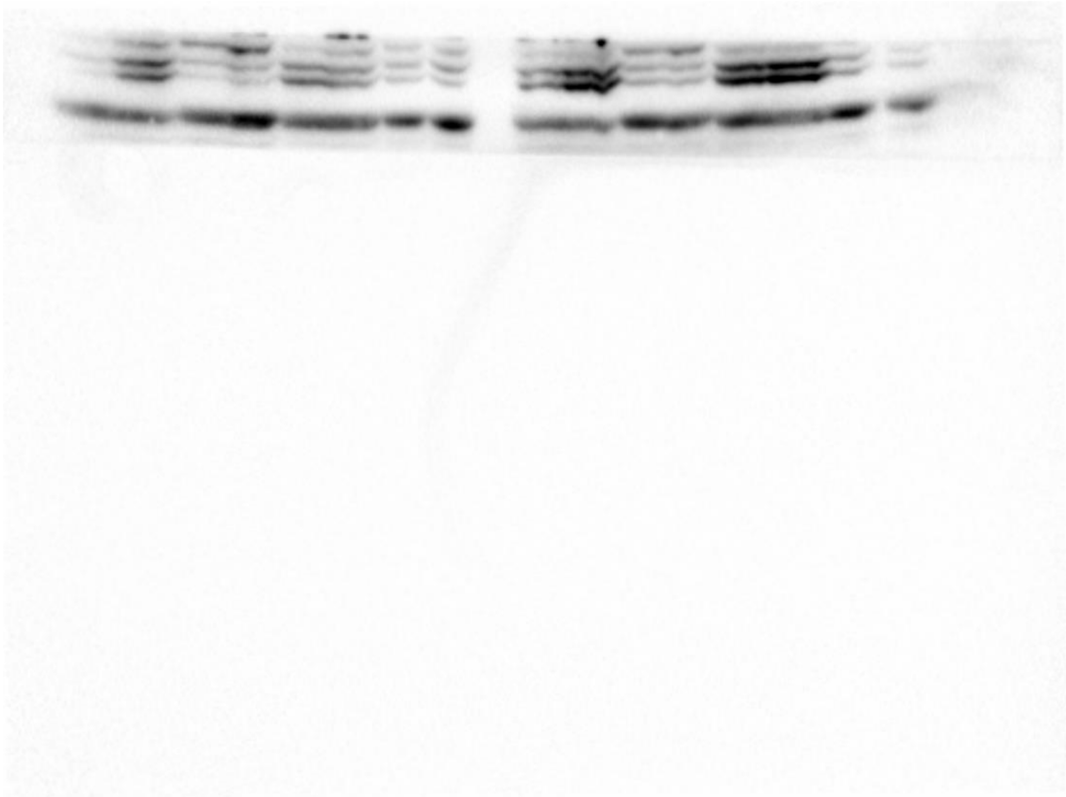

Yap1

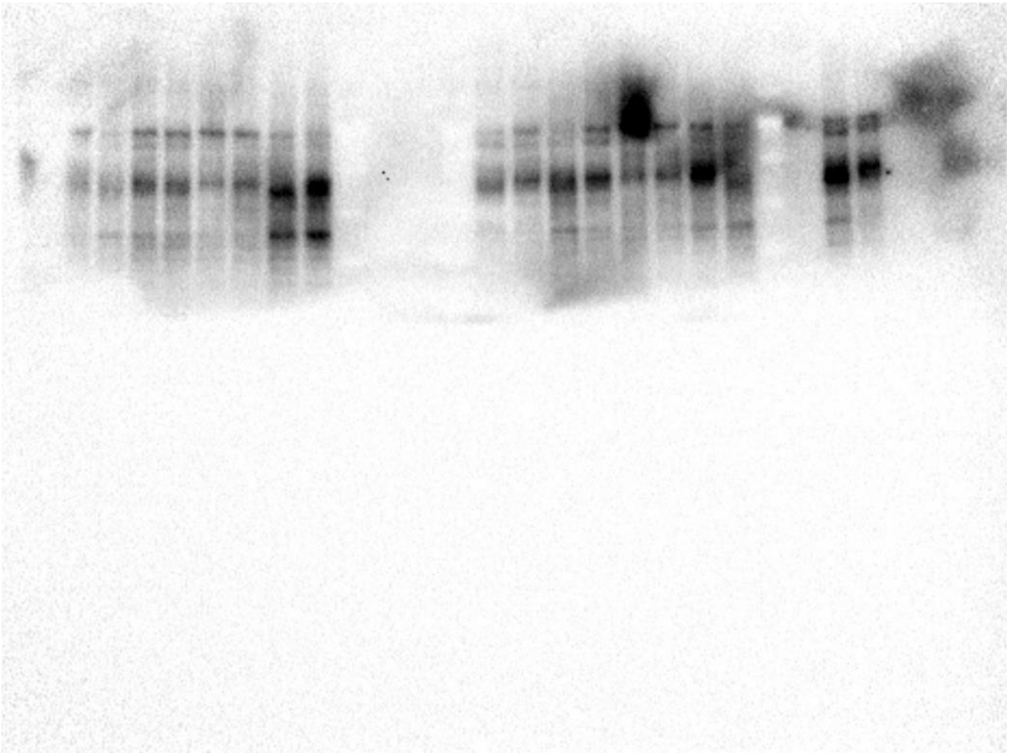

P-Taz

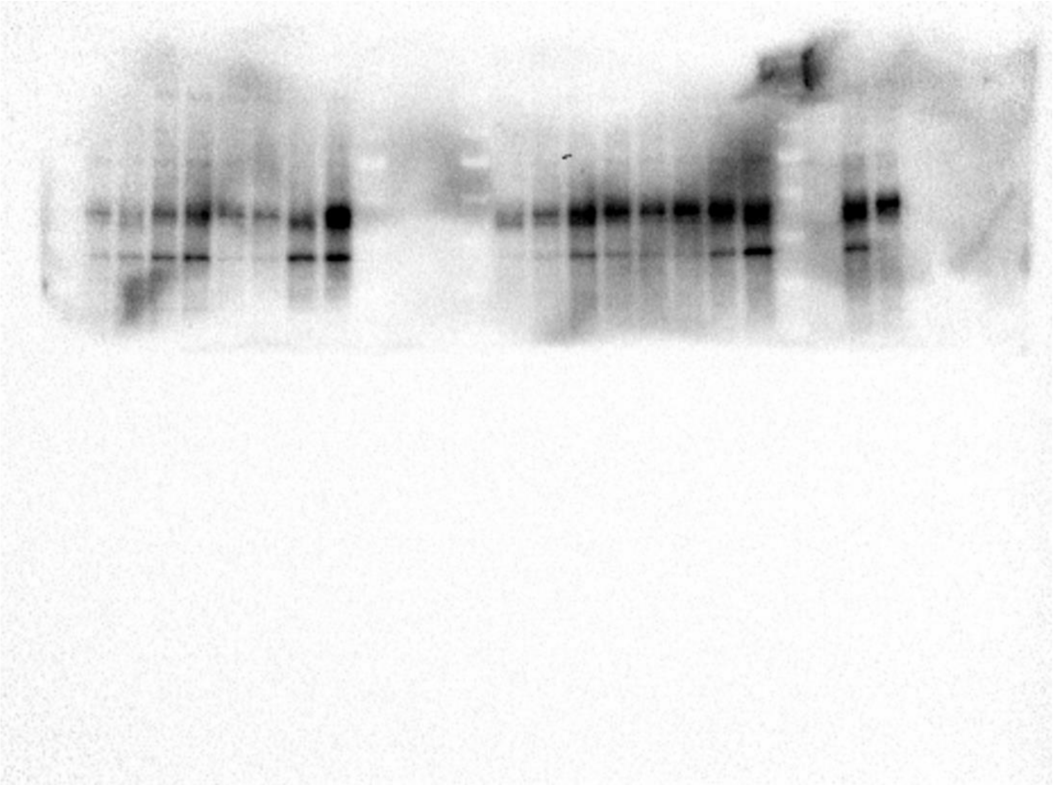

Taz

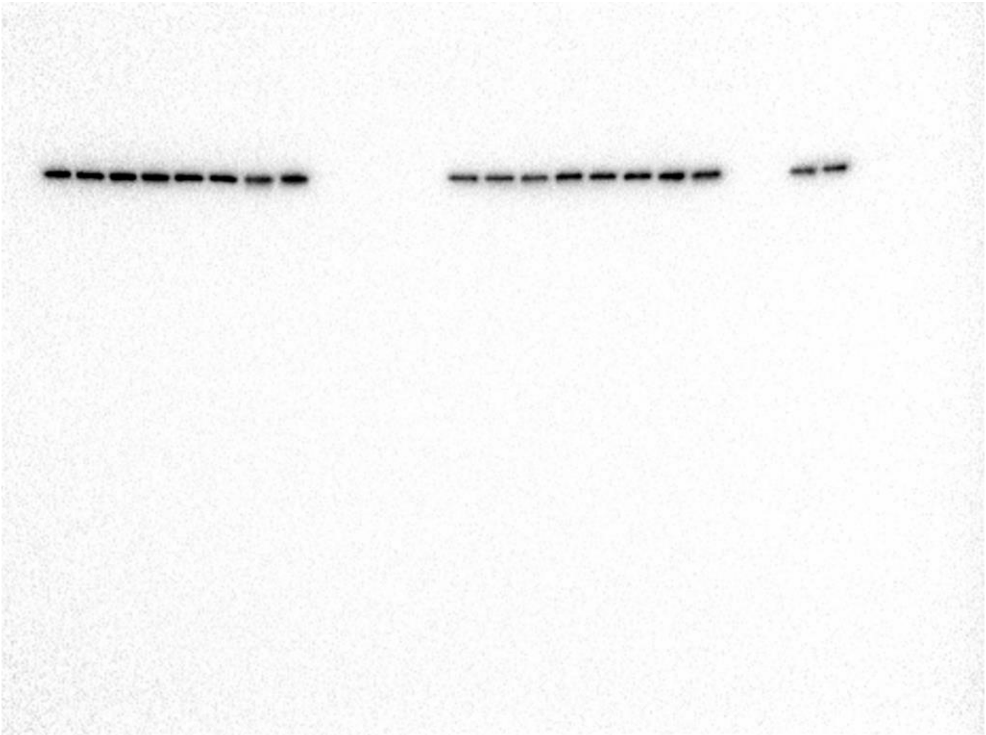

B-Actin

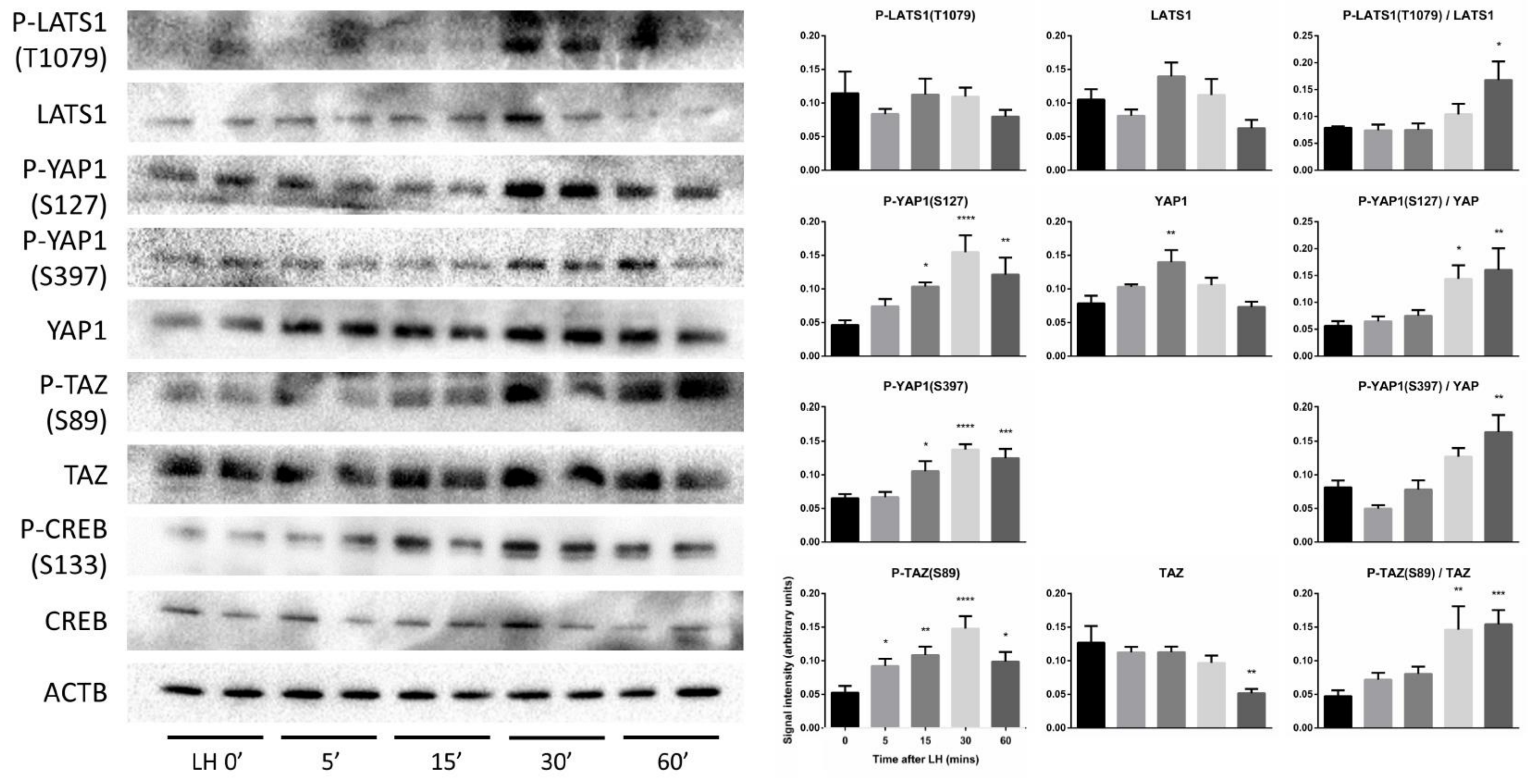
P-LATS1 (T1079)
LATS1
P-YAP1 (S127)
P-YAP1 (S397)
YAP1
P-TAZ (S89)
TAZ
P-CREB (S133)
CREB
ACTB
LH 0'
5'
15'
30'
60'
P-LATS1(T1079)
LATS1
P-LATS1(T1079) / LATS1
P-YAP1(S127)
YAP1
P-YAP1(S127) / YAP
P-YAP1(S397)
P-YAP1(S397) / YAP
P-TAZ(S89)
TAZ
P-TAZ(S89) / TAZ
Signal intensity (arbitrary units)
Time after LH (mins)

FIGURE 2

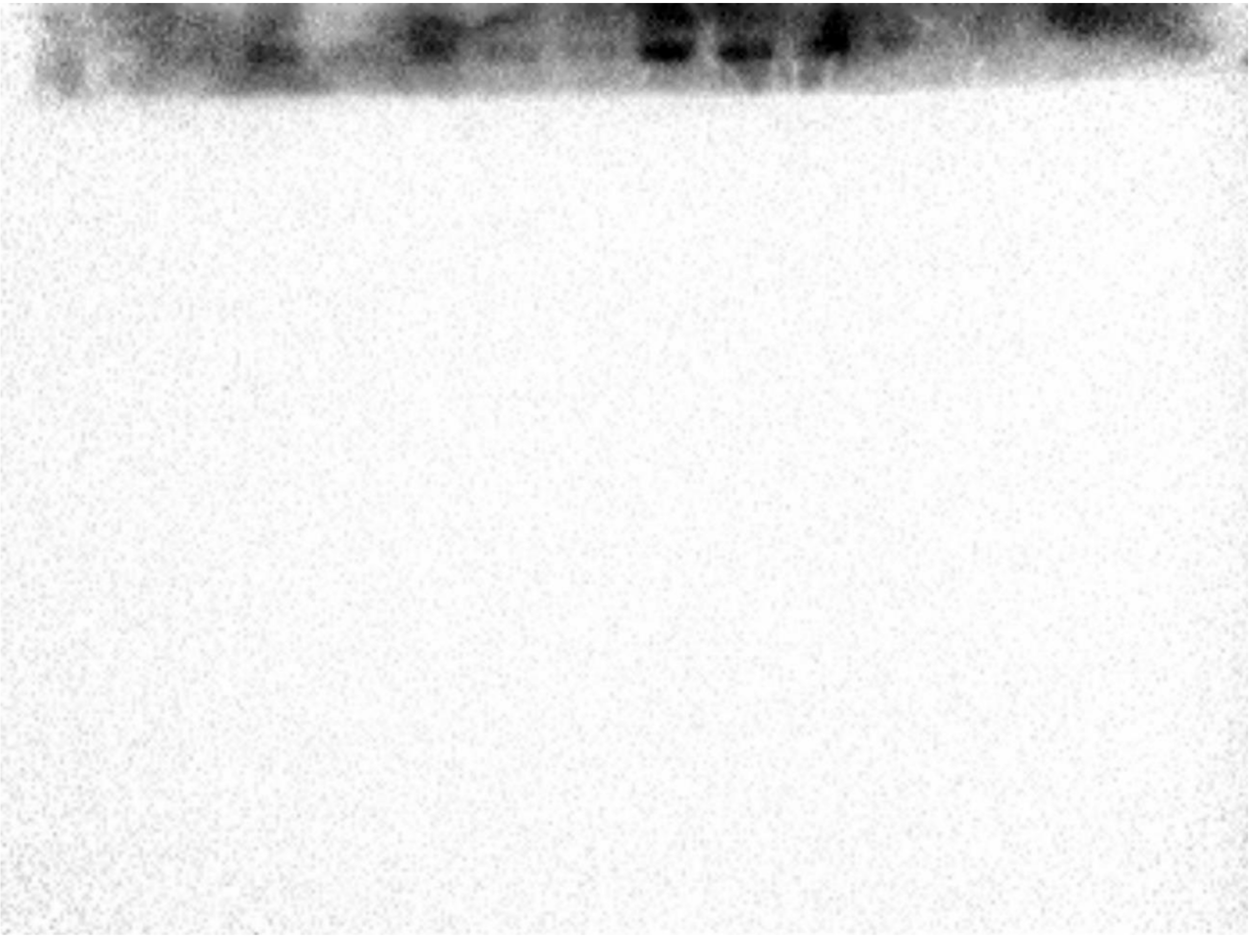

P-lats1

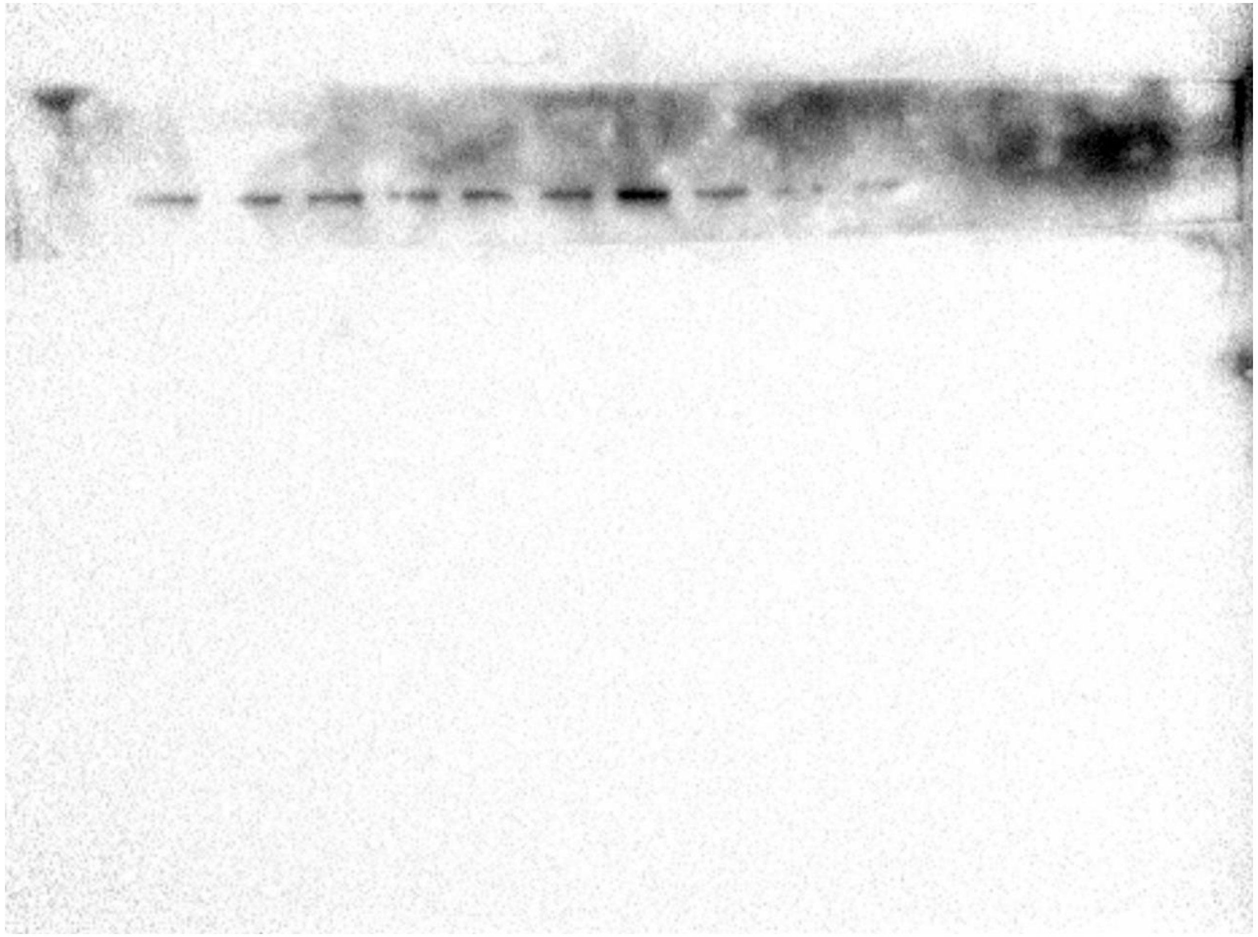

Lats1

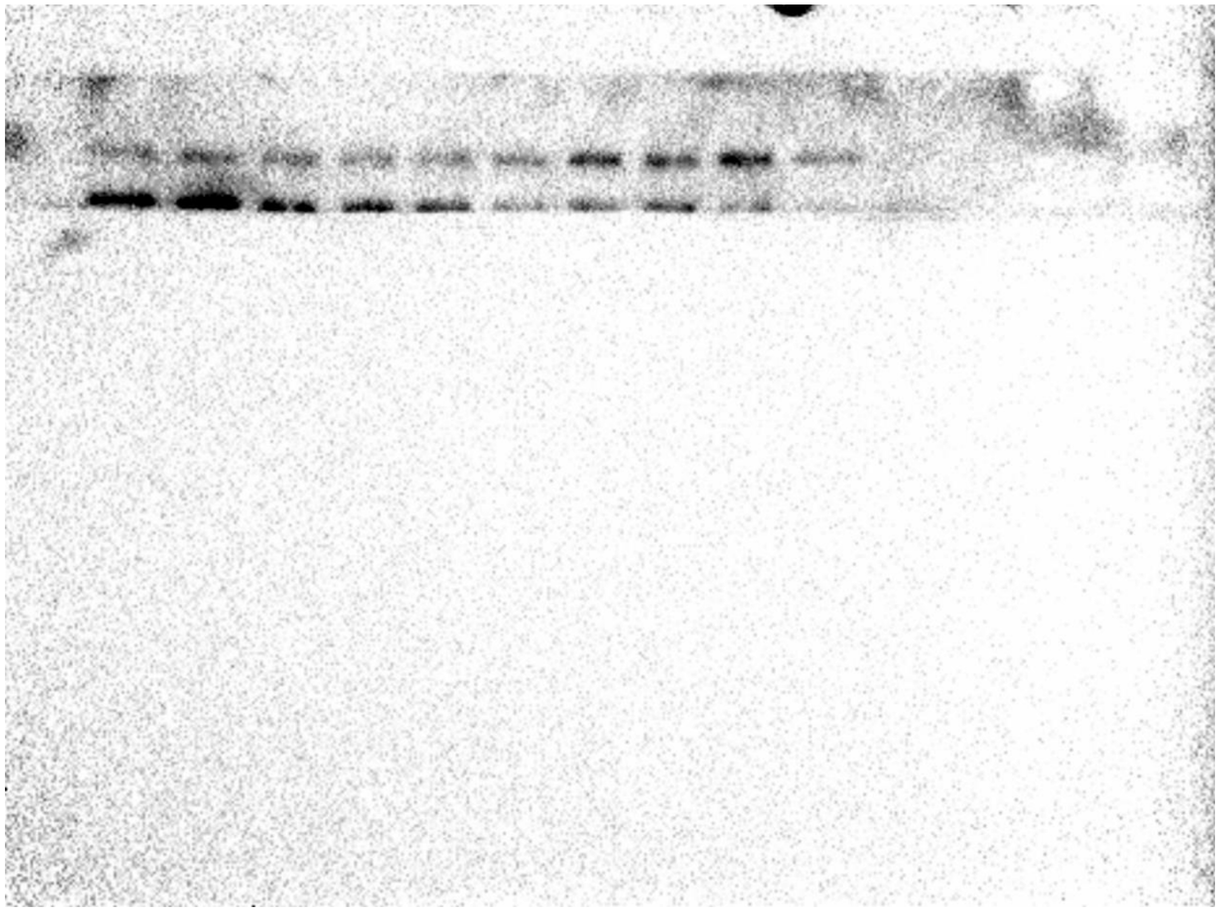

P-Yap1 397

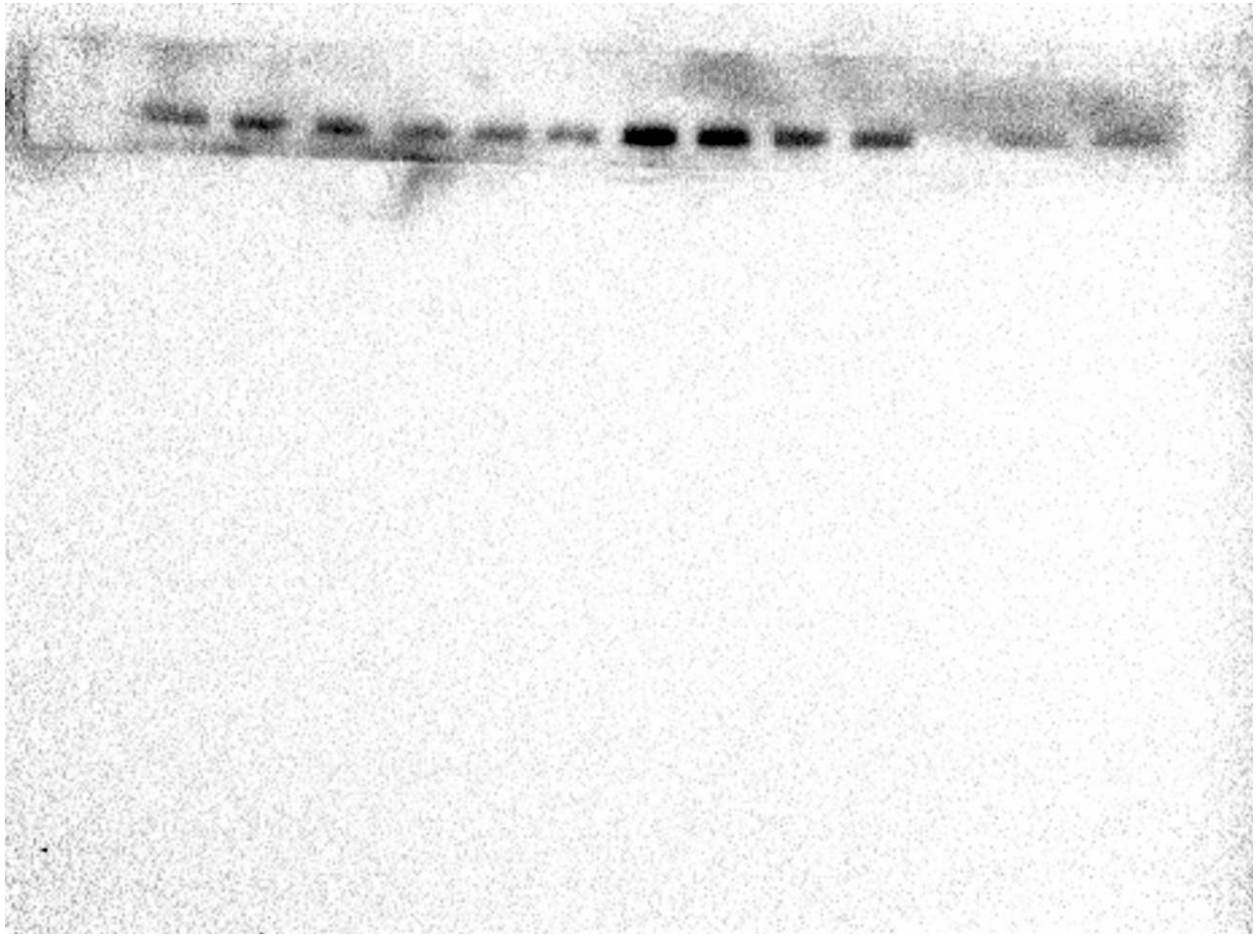

P-Yap1 127

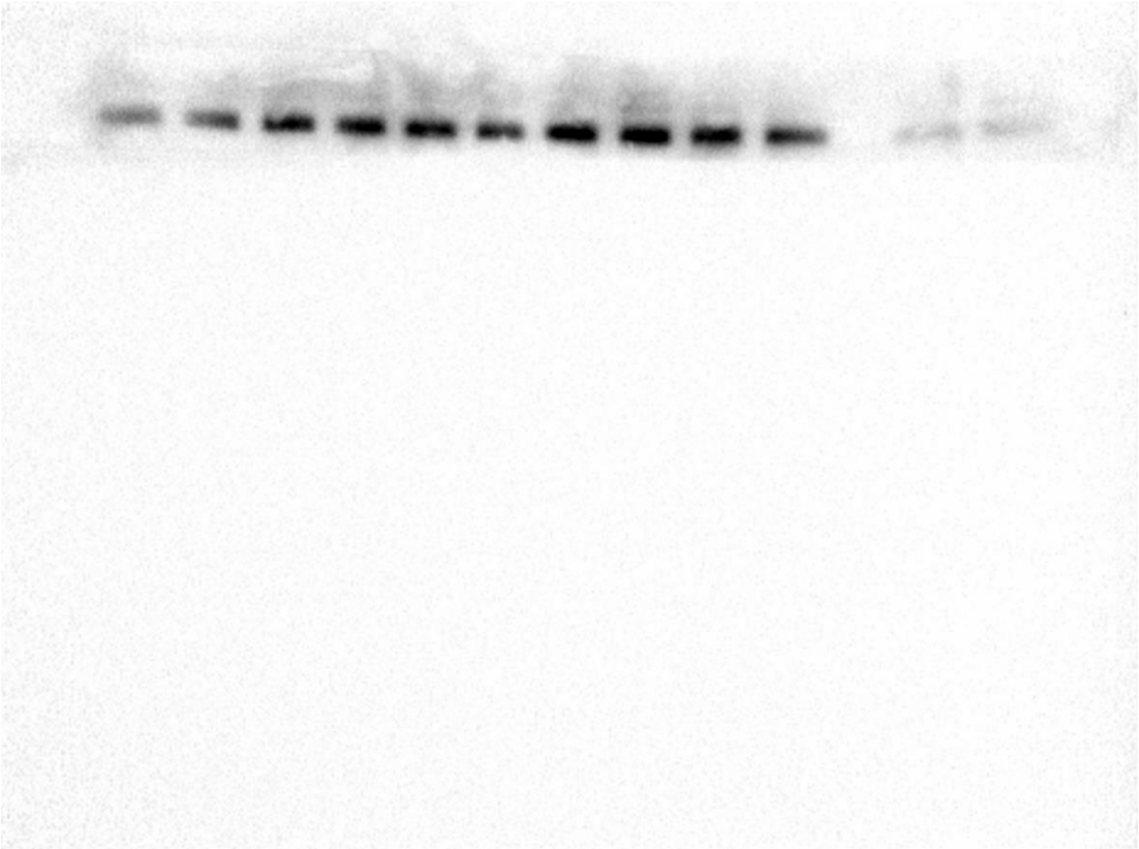

Yap1

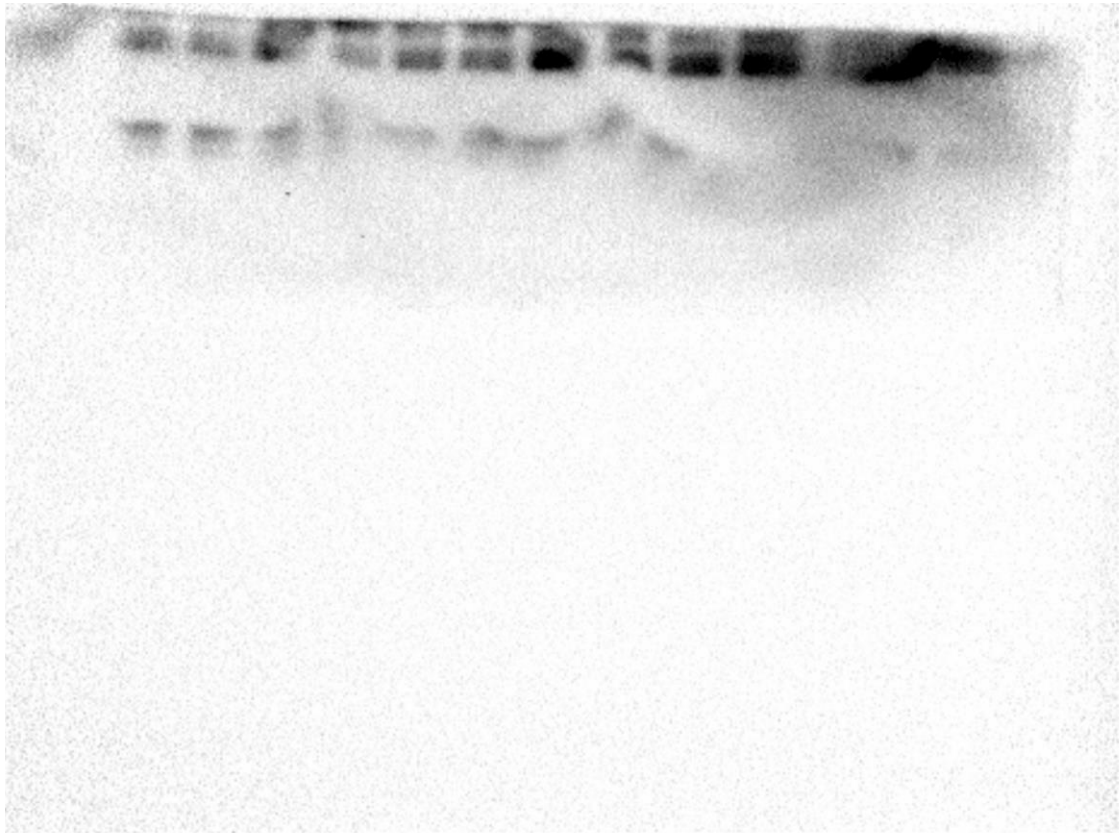

P-Taz

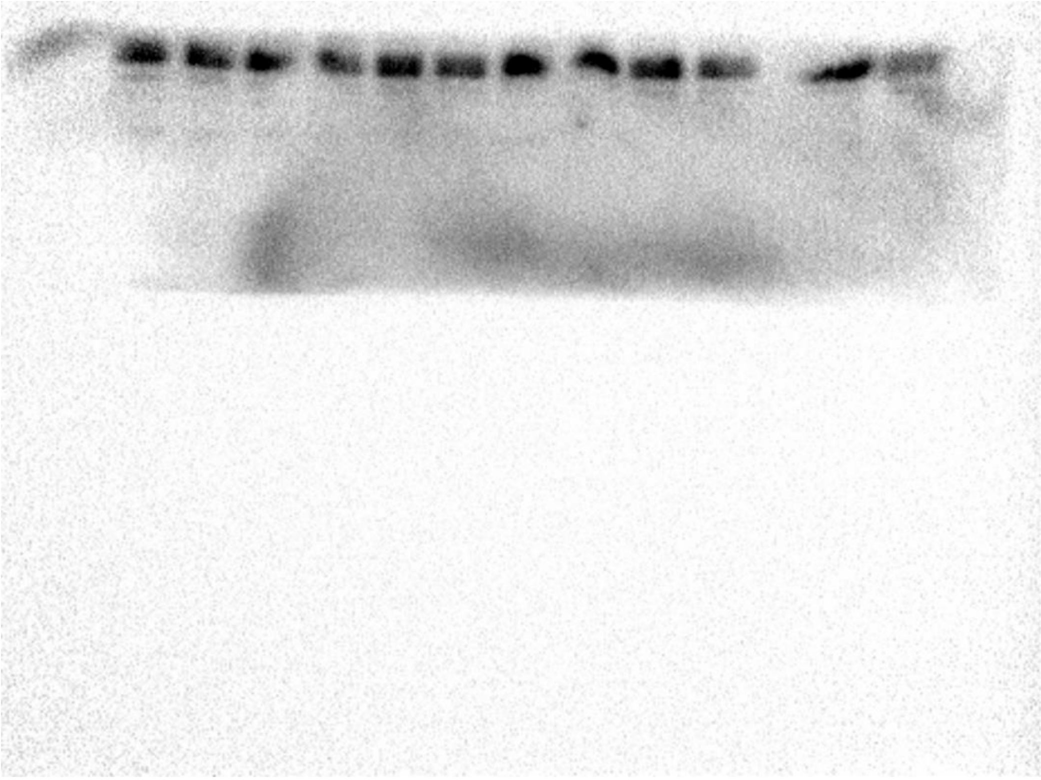

Taz

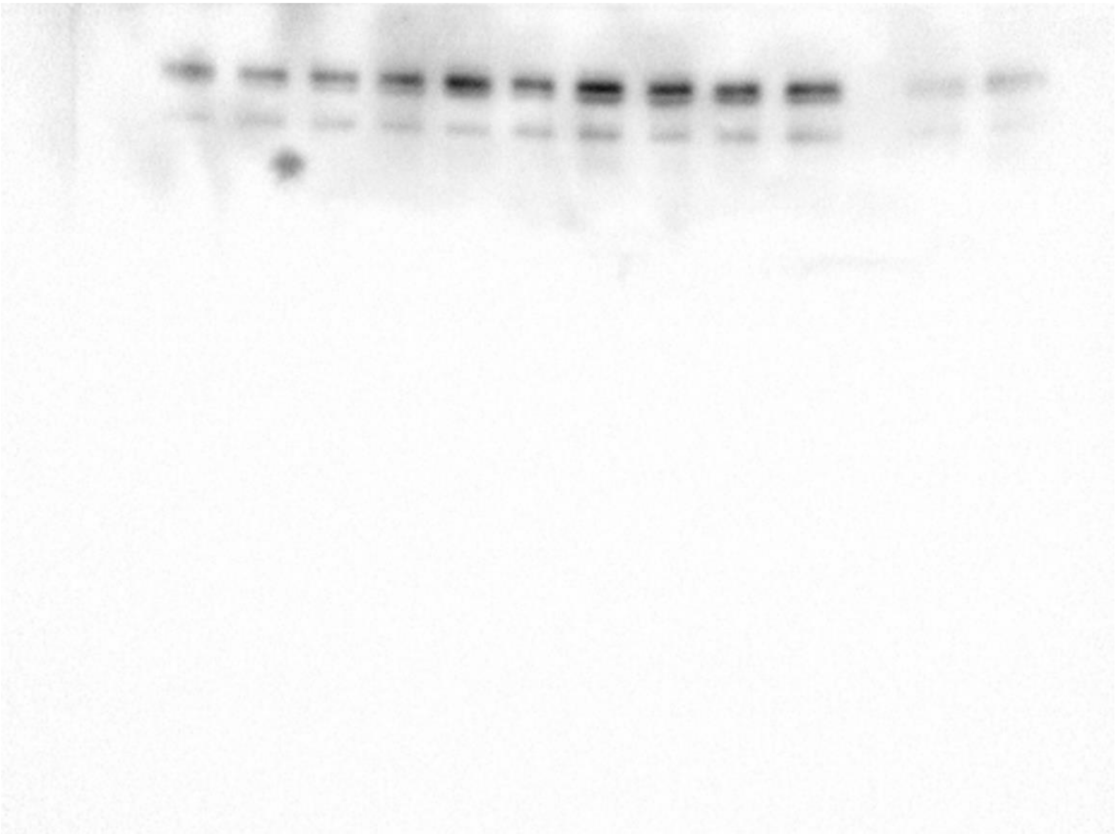

P-Creb

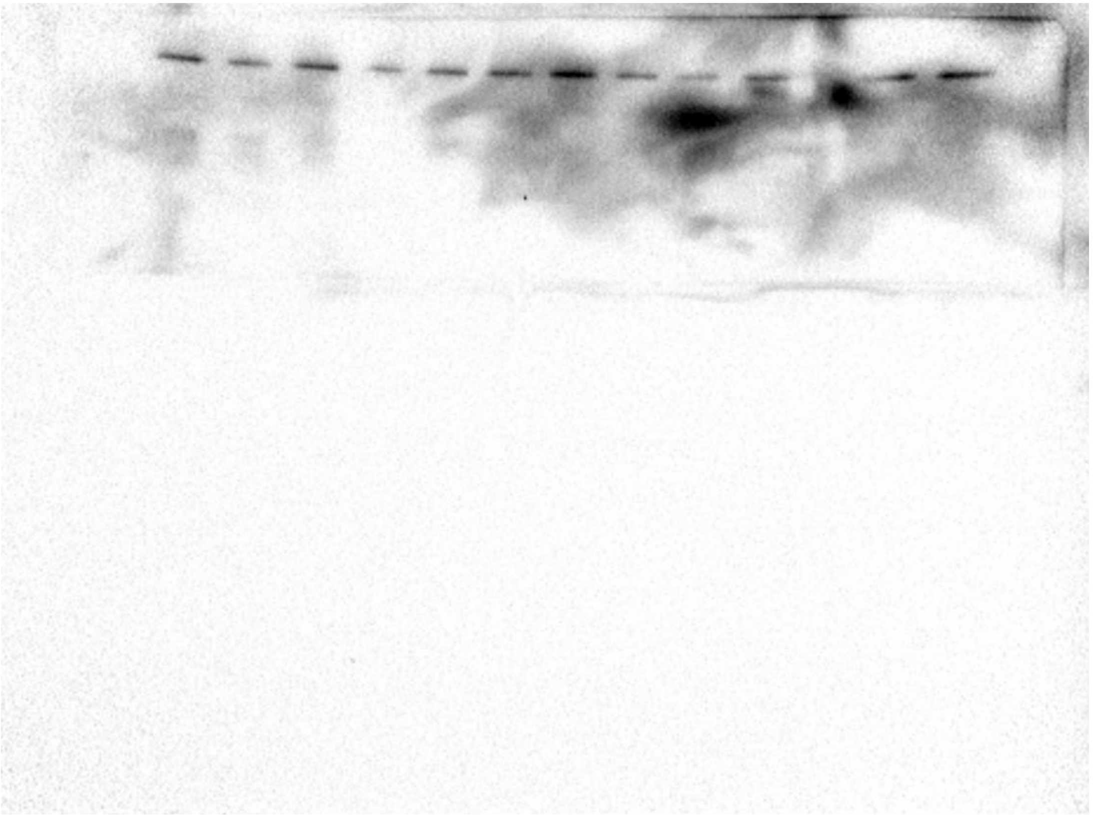

Creb

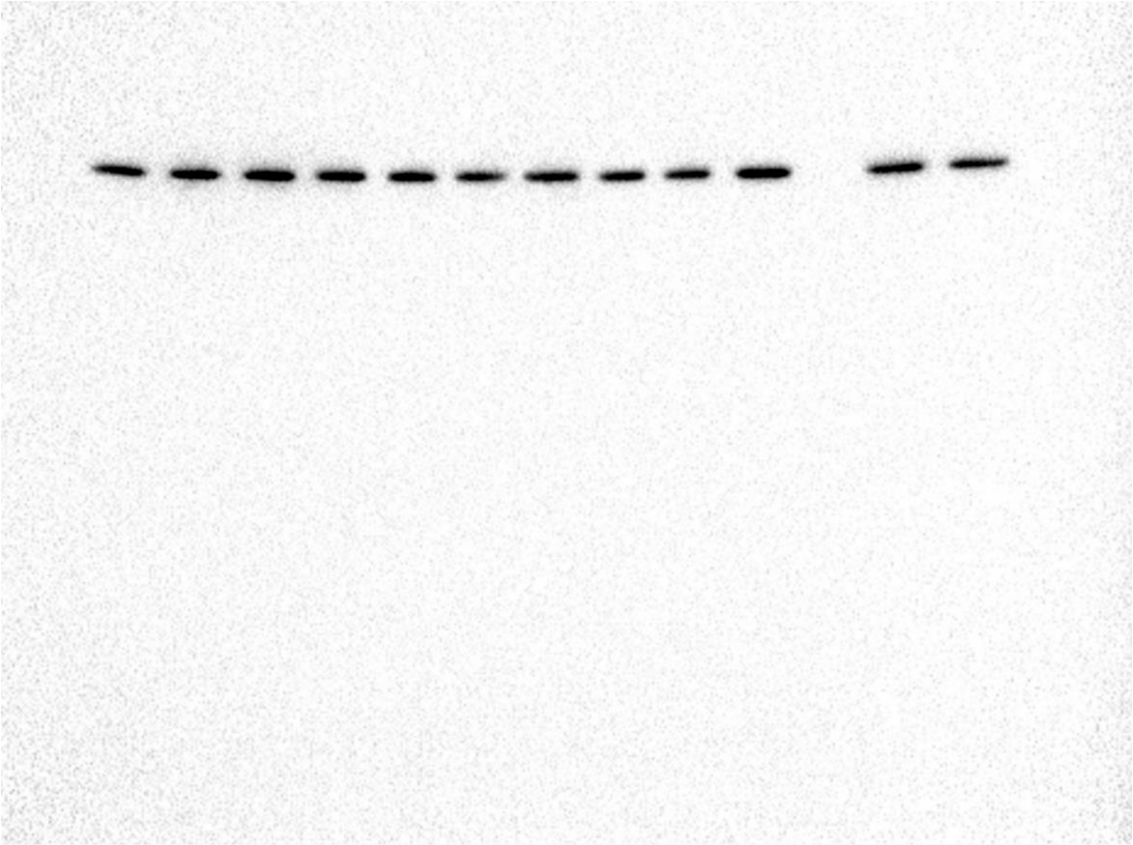

B-Actin

FIGURE 3

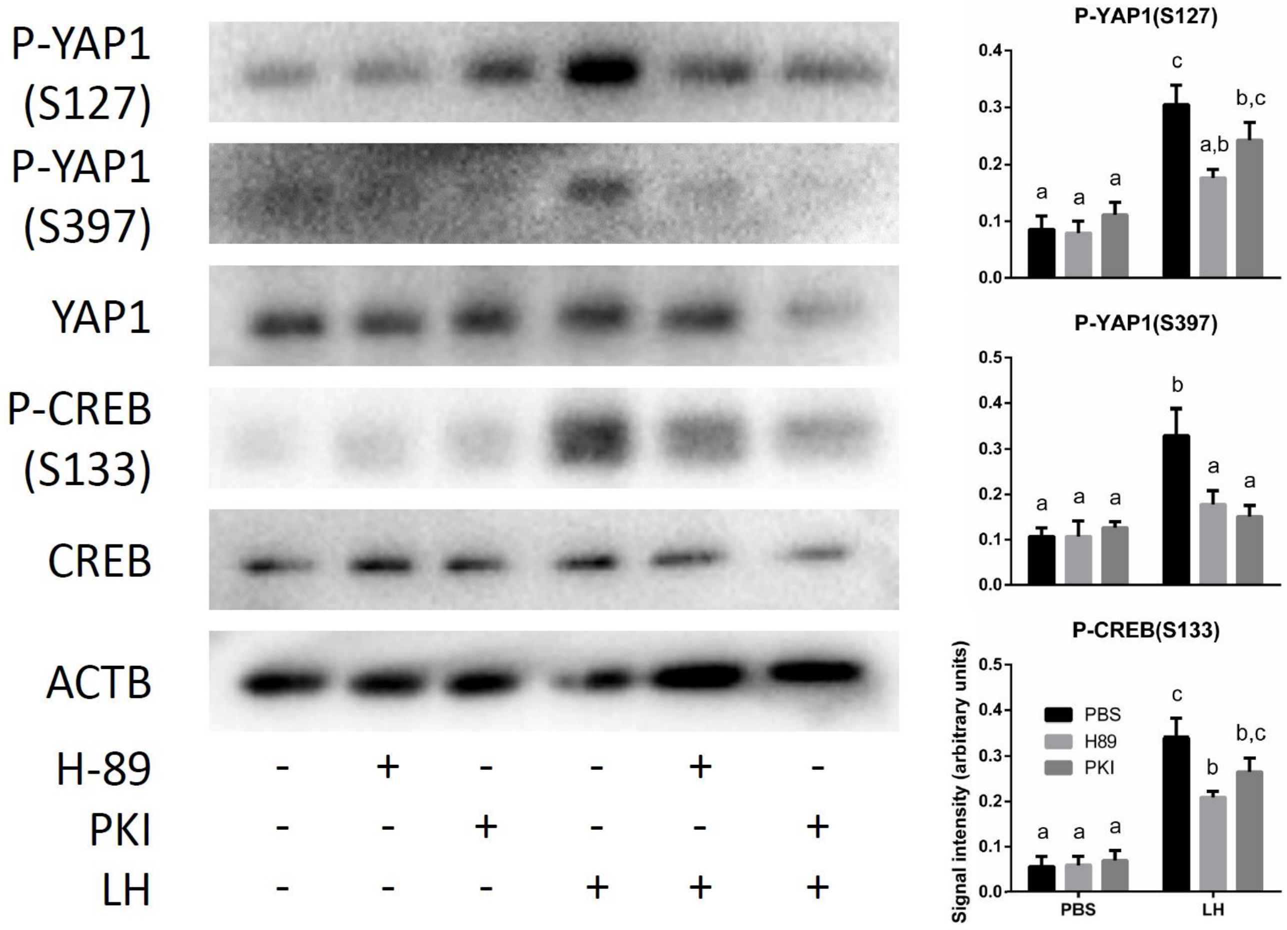
P-YAP1 (S127)
P-YAP1 (S397)
YAP1
P-CREB (S133)
CREB
ACTB
H-89 - + - - + -
PKI - - + - - +
LH - - - + + +
P-YAP1(S127)
P-YAP1(S397)
P-CREB(S133)
Signal intensity (arbitrary units)
PBS
H89
PKI
PBS
LH

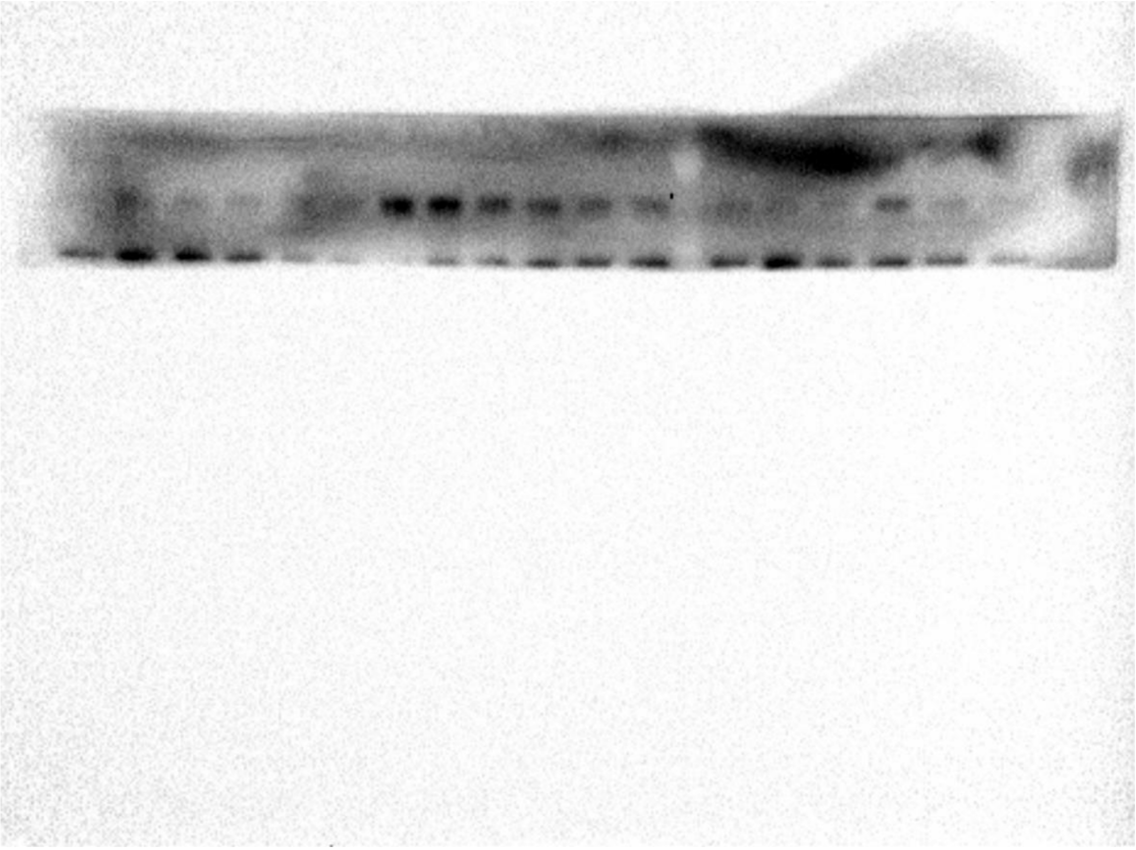

P-Yap1 397

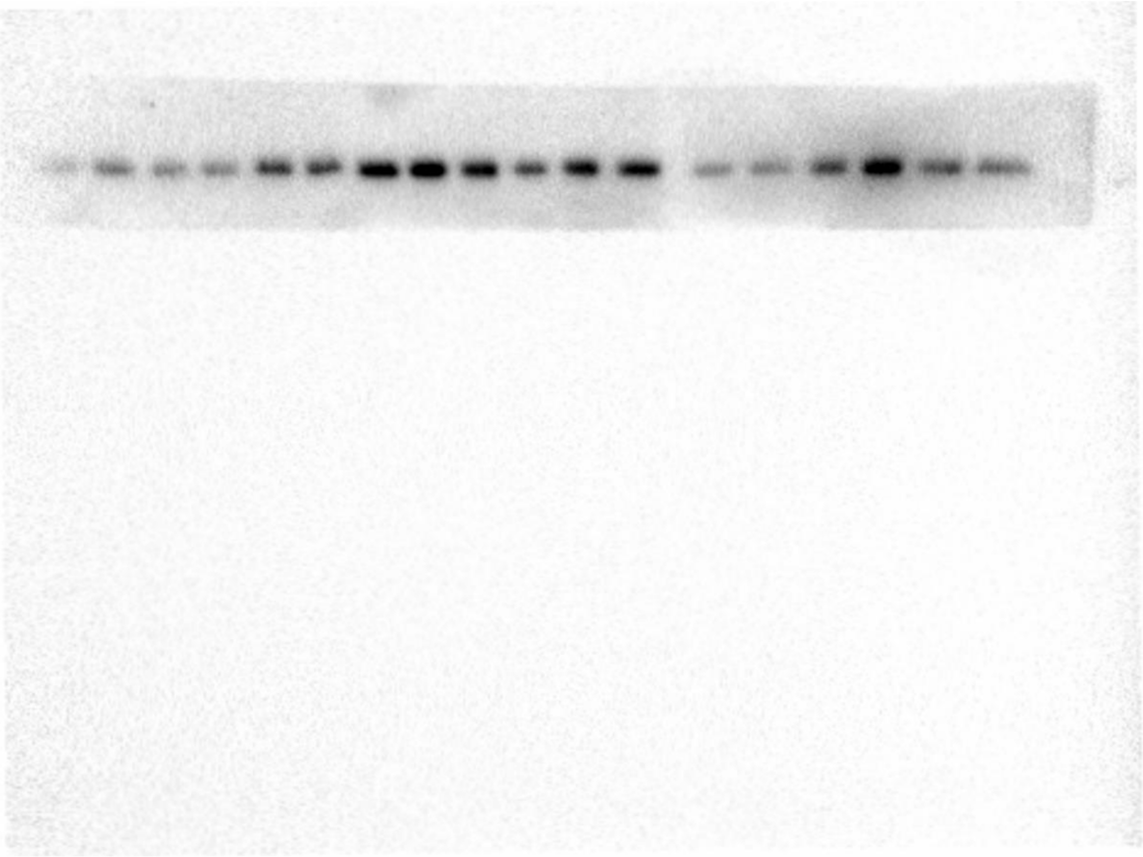

P-Yap1 127

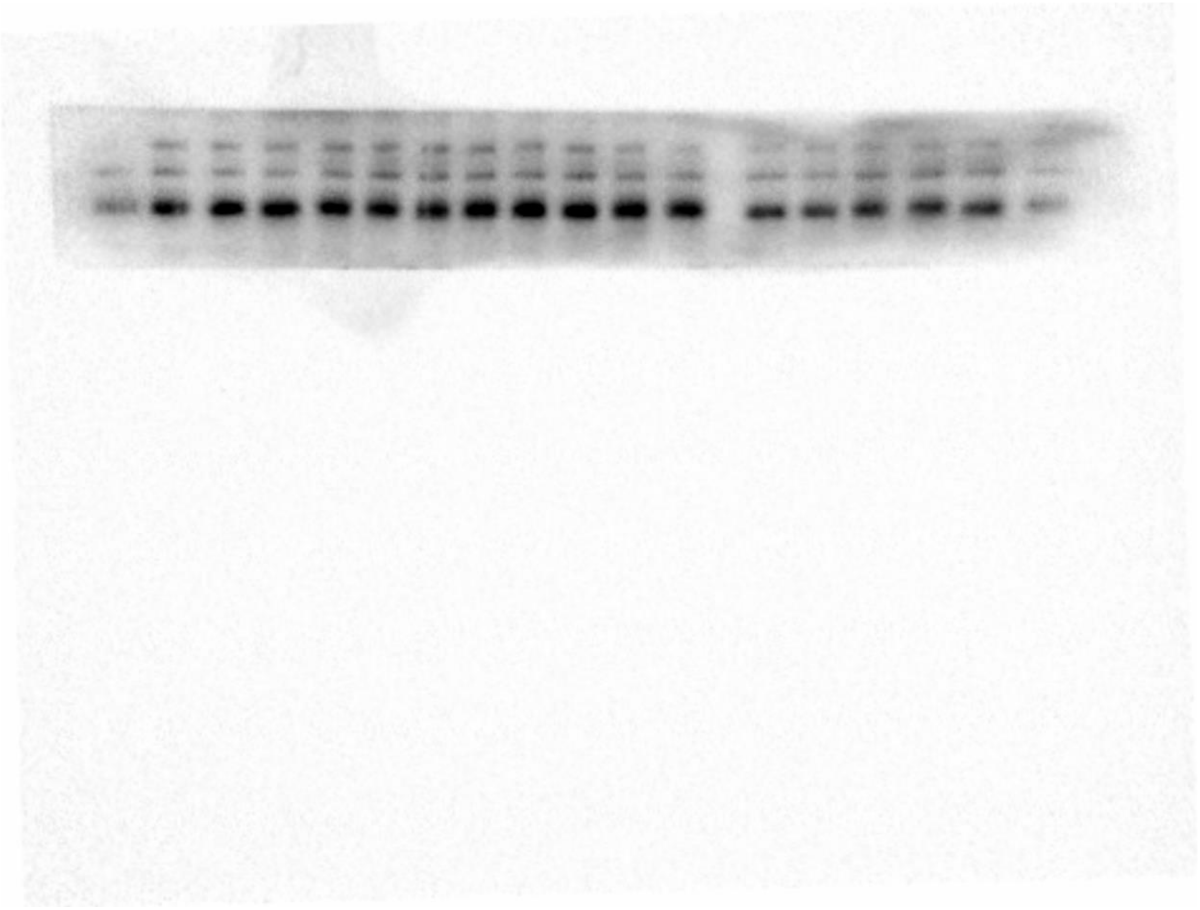

Yap1

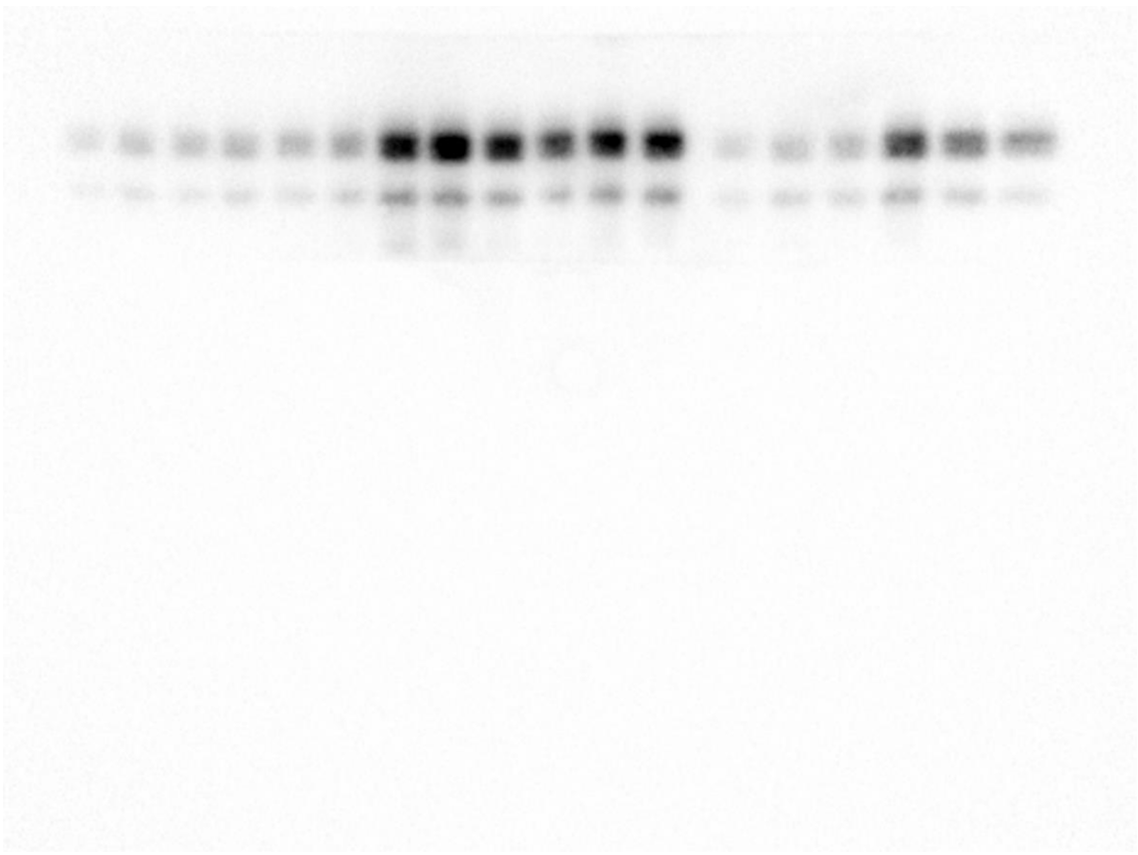

P-Creb133

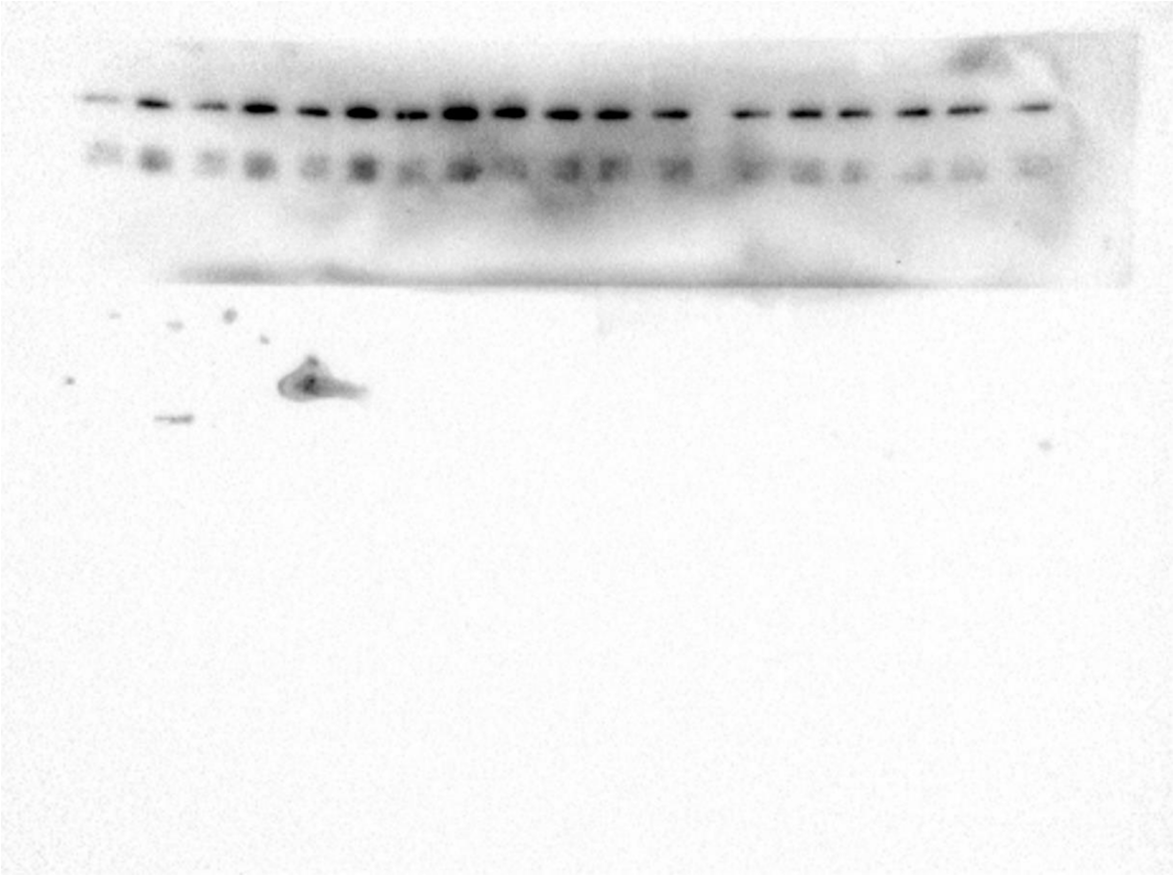

Creb

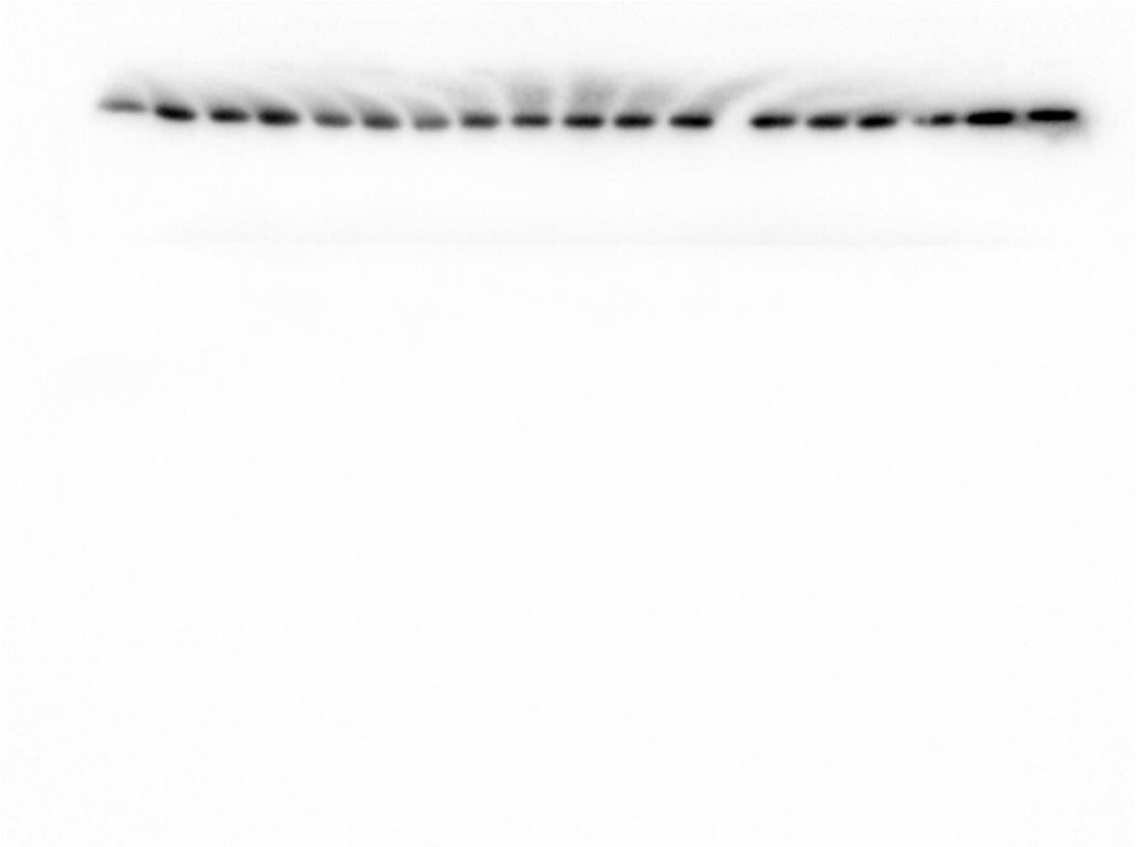

B-Actin
